# Polydatin Delivery Systems and Nanomedicine: Pharmacology, Preclinical Evidence, and Translation

**DOI:** 10.3390/pharmaceutics18091134

**Published:** 2026-09-09

**Authors:** Xiaoya Li, Lingling Li, Yongfang Yuan, Changxin Sun, Yajie Wang, Xuefei Wang, Yixuan Feng, Min Wu, Longtao Liu

**Affiliations:** 1 Department of Cardiology, Guang’anmen Hospital, China Academy of Chinese Medical Sciences, Beijing 100053, China; lixiaoya0303@foxmail.com (X.L.);; 2 State Key Laboratory of Traditional Chinese Medicine Syndrome/National Clinical Research Center for Chinese Medicine Cardiology, Xiyuan Hospital, China Academy of Chinese Medical Sciences, Beijing 100091, China; 3 School of Clinical Medicine, Beijing University of Chinese Medicine, Beijing 100029, China

**Keywords:** polydatin, nanomedicine, drug delivery, pharmacokinetics, targeted delivery

## Abstract

**Background:** Polydatin has shown anti-inflammatory, antioxidant, and cytoprotective effects in preclinical studies. Its development is limited by low oral bioavailability, extensive metabolism, and poorly defined tissue exposure. **Objectives:** This review summarizes the pharmacology and delivery systems of polydatin, distinguishes nano from non-nano formulations, and examines the evidence for improved exposure, tissue delivery, safety, and clinical translation. **Methods:** Relevant studies were identified through a structured PubMed search and assessed by formulation type, administration route, comparator, and reported endpoints. **Results:** Delivery strategies include liposomes, polymeric and polysaccharide nanoparticles, lipid-based formulations, inclusion complexes, solid-state systems, hydrogels, local matrices, and targeted systems. Only a few oral formulations have been compared directly with free polydatin using quantitative pharmacokinetic measurements. Many injectable and local studies report release, cellular uptake, fluorescence distribution, or pharmacodynamic effects, but these findings do not by themselves demonstrate improved bioavailability or drug-specific targeting. Evidence on repeat-dose toxicity, immunogenicity, carrier clearance, and manufacturing consistency remains limited. No clinical study of a polydatin nanocarrier was identified. **Conclusions:** Further development requires appropriate free-polydatin controls, measurement of polydatin and its metabolites, exposure–response analysis, fuller safety assessment, and reproducible product characterization.

## 1. Introduction

Polydatin, also known as piceid, is a naturally occurring stilbenoid glucoside and a characteristic constituent of *Reynoutria japonica* (syn. *Polygonum cuspidatum*) [1]. As a purified compound with defined composition, polydatin permits controlled dosing and clearer attribution of pharmacological and formulation effects than crude botanical extracts [2,3]. Pharmacological studies have examined its effects in cardiovascular and cerebrovascular injury, metabolic and renal disorders, inflammatory and fibrotic diseases, and tissue repair [4,5,6,7]. Across these settings, polydatin has been associated with redox regulation, mitochondrial and endoplasmic reticulum homeostasis, innate immune and inflammasome signaling, regulated cell death, autophagy, and mitophagy [8,9,10,11,12]. Many of these mechanisms operate within specific tissues, cell populations, and intracellular compartments, making tissue, cellular, and intracellular exposure relevant to the magnitude and duration of the observed pharmacological responses.

Achieving exposure sufficient to engage these mechanisms depends on the route of administration. Oral dosing is influenced by dissolution, intestinal transport, hydrolysis, phase-II metabolism, and systemic clearance, which contribute to low bioavailability and variable systemic exposure [13,14,15,16]. Intravenous administration avoids gastrointestinal processing but remains subject to stability in circulation, mononuclear phagocyte system clearance, tissue distribution, cellular uptake, and intracellular release [17,18]. Local administration instead depends on residence at the application site, tissue penetration, release duration, carrier degradation, and local tolerance [19,20]. Although the dominant barrier differs by route, each can limit the concentration or duration of polydatin exposure at relevant sites of action.

**Figure 1:**
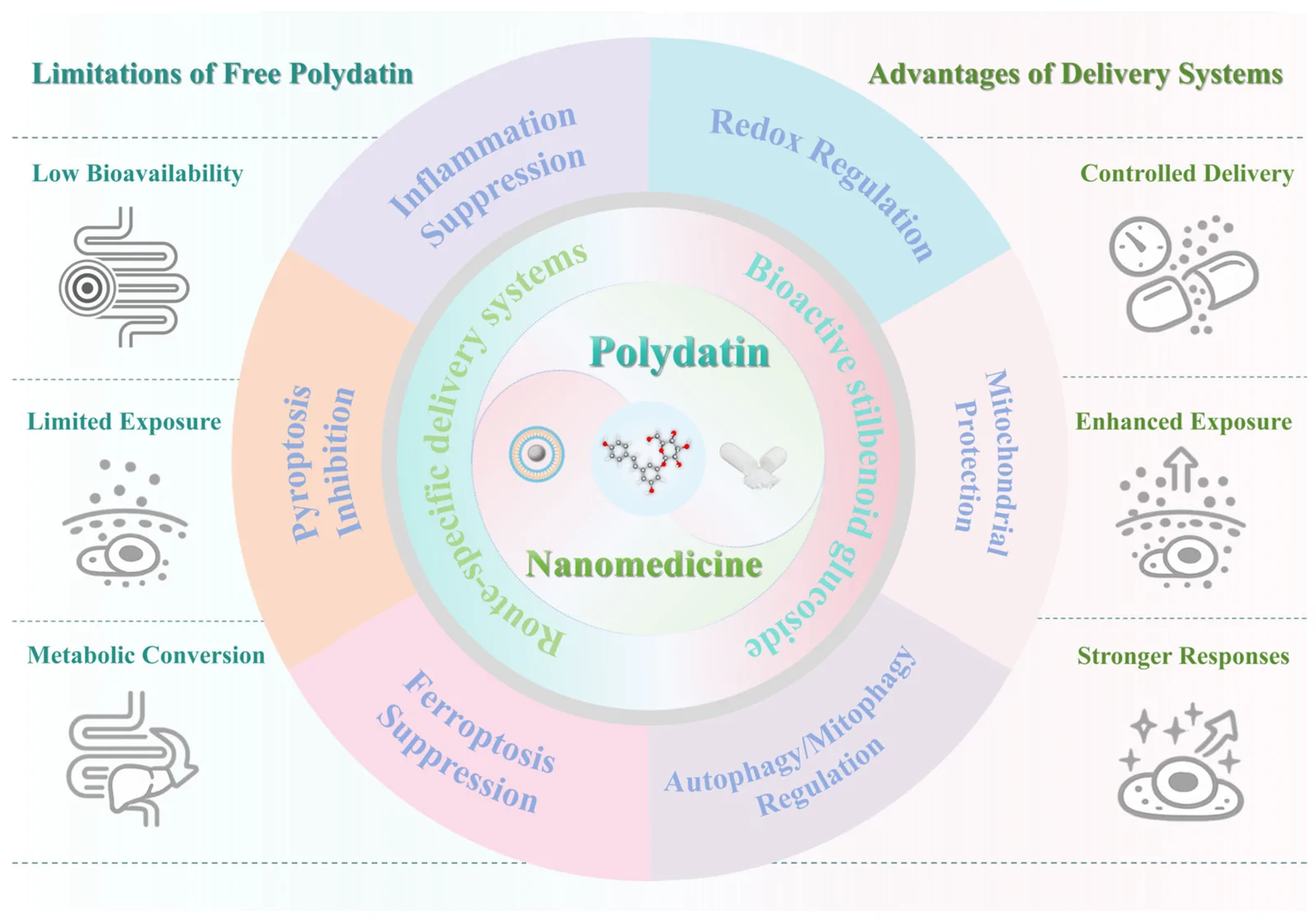
Overview of polydatin pharmacology, exposure limitations, and delivery systems. Polydatin has multiple reported pharmacological actions, but low oral bioavailability, metabolic conversion, and insufficient exposure at relevant tissue and cellular sites may limit their magnitude or duration. Delivery systems have been investigated to control drug release, modify systemic exposure and distribution to target sites, enhance cellular uptake, and prolong local residence. In selected disease models, some formulations produced stronger pharmacodynamic responses than dose-matched free polydatin.

Polydatin delivery research now spans oral, injectable, and local formulations designed to address route-specific limitations in exposure and tissue residence [21,22,23,24,25]. Unlike earlier reviews centered on general pharmacology, pharmacokinetics, or selected disease areas [4,6], this review asks whether formulation strategies change exposure, distribution, or efficacy and distinguishes nanocarriers from non-nano enabling systems. It thereby defines which approaches have demonstrated delivery value and which still lack the pharmacokinetic, safety, quality, or clinical evidence required for translation (Figure 1).

### Literature Search and Evidence Selection

This critical narrative review was informed by a structured PubMed search conducted on 7 July 2026 and updated on 26 August 2026. Search terms combined polydatin/piceid with concepts related to delivery systems, formulation and release, solid-state and local-delivery approaches, pharmacokinetics, bioavailability, biodistribution, analytical methods, and delivery-relevant mechanisms. The searches retrieved 590 record occurrences; after removal of 156 duplicate occurrences, 434 unique records underwent title/abstract screening. Original experimental studies were eligible when polydatin/piceid was a defined payload or formulation component and they reported delivery, formulation, exposure, distribution, or prespecified mechanistic data. Eligible non-nano formulation strategies were included as contextual comparators and analyzed separately from nanocarriers. Records without a defined polydatin intervention or relevant endpoint, carrier-only or experimentally unvalidated computational studies, non-primary reports, corrections, retractions, and botanical or food-chemistry records unrelated to delivery were excluded. Screening retained 157 records: 38 core delivery/formulation, 31 supporting formulation/pharmacokinetic, and 88 mechanistic/background records. Complete search strings, eligibility criteria, study-level classifications [26,27], exclusion reasons, and the adapted PRISMA-style flow are provided in Supplementary Tables S1–S4 and Figure S1; the complete study-level mechanistic evidence matrix is provided in Supplementary Table S5.

## 2. Polydatin Properties and Exposure Barriers

### 2.1. Chemical Identity and Biotransformation

**Figure 2:**
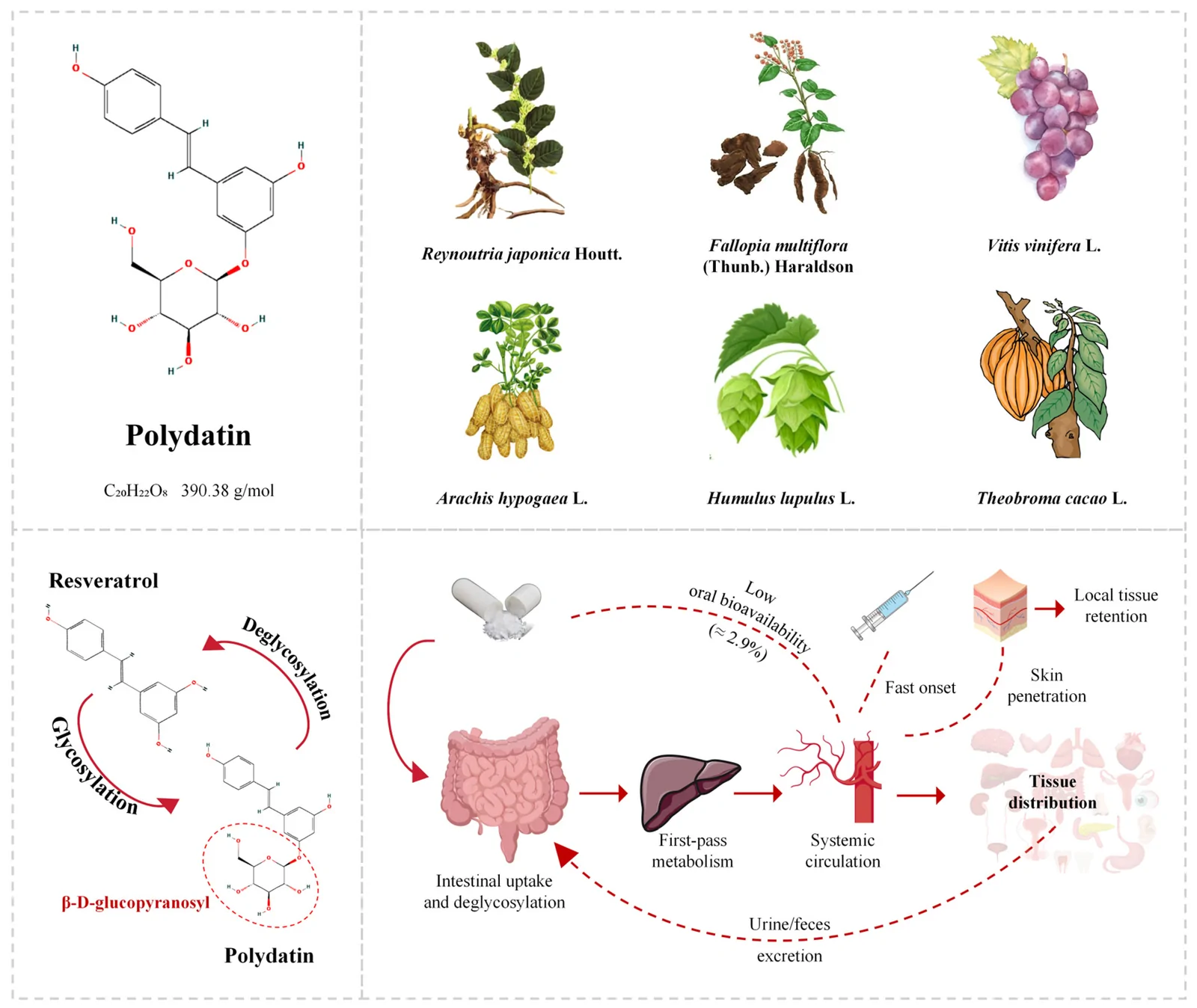
Chemical identity, natural sources, biotransformation, and pharmacokinetic disposition of polydatin. Polydatin is a stilbenoid glucoside with the molecular formula C_20_H_22_O_8_ and a molecular weight of 390.38 g/mol. It occurs in *Reynoutria japonica*, *Fallopia multiflora*, grapes, peanuts, hops, and cocoa. Polydatin can undergo deglycosylation to form resveratrol, whereas glycosylation of resveratrol generates polydatin through addition of a β-D-glucopyranosyl residue. After oral administration, polydatin undergoes intestinal uptake and deglycosylation, followed by first-pass metabolism and entry into the systemic circulation, with an absolute oral bioavailability of approximately 2.9%. Intravenous administration provides rapid systemic exposure, while formulation-assisted local delivery promotes skin penetration and local tissue retention. Polydatin and its metabolites are subsequently distributed to tissues and eliminated through urine and feces.

Polydatin, also known as piceid, is the 3-O-β-D-glucopyranoside of resveratrol. It has a molecular formula of C_20_H_22_O_8_ and a molecular weight of 390.38 g/mol and consists of a stilbene scaffold with a β-D-glucopyranosyl residue linked through the phenolic hydroxyl group at C-3 (Figure 2) [15,28]. It is a characteristic stilbenoid constituent of the rhizomes and roots of *Reynoutria japonica* Houtt. [syn. *Polygonum cuspidatum* Siebold & Zucc.; *Fallopia japonica* (Houtt.) Ronse Decr.] and has also been detected in grapes, red wine, peanuts, hop cones or pellets, and cocoa- or chocolate-containing products [1,15]. Both trans- and cis-isomers exist, although trans-polydatin is the form most commonly investigated. The configuration is analytically relevant because irradiation of trans-polydatin in solution can induce conversion to the cis-isomer [29].

The glucose residue changes several properties of the stilbene structure. Relative to resveratrol, polydatin has greater molecular size, polarity, and hydrogen-bonding capacity, together with different crystal packing, aqueous dissolution, membrane interaction, and susceptibility to transporter- and glycosidase-mediated processes. These differences affect absorption and metabolism and preclude treating polydatin and resveratrol as interchangeable compounds. Polydatin is therefore better regarded as a distinct stilbenoid glucoside whose biological disposition includes, but is not limited to, conversion to resveratrol [13,28].

The glycosidic bond is susceptible to microbial and enzymatic hydrolysis. Fermentation of *P. cuspidatum* with *Aspergillus oryzae* increased resveratrol formation through conversion of piceid, while cell extracts from several *Bifidobacterium* and *Lactobacillus* strains also catalyzed deglycosylation [30,31]. Although these experiments used fermentation or cell-extract systems, they establish that polydatin can serve as a substrate for microbial β-glucosidase activity. Intestinal epithelial studies further show that trans-piceid can undergo transport and deglycosylation concurrently, followed by glucuronidation of the resulting resveratrol [13]. Consequently, biological samples obtained after polydatin administration may contain intact polydatin, resveratrol, and conjugated forms of both compounds, each with potentially different distribution and biological activity.

### 2.2. Physicochemical and Pharmacokinetic Barriers

The physicochemical limitations of polydatin cannot be described simply as generalized chemical instability. In a preformulation study, crystalline trans-piceid showed negligible degradation under accelerated heat and humidity, ambient fluorescent light, and International Council for Harmonisation photostability conditions; the chromatographic main-peak area remained between 98.04% and 99.35% across the 12-week study [32]. Solid-state form nevertheless affects pharmaceutical behavior. An anhydrous polymorph, a trihydrate, and an amorphous form have been characterized; the two crystalline forms remained stable during the reported storage study, whereas the amorphous material converted rapidly to the anhydrous form. After an equal oral dose in rats, the trihydrate showed faster absorption and approximately 1.5-fold greater exposure than the anhydrous polymorph [28]. Together with the solution-state photoisomerization described above, these data indicate that stability and exposure depend on physical form and experimental conditions rather than on a single intrinsic property.

At the intestinal epithelium, transport occurs alongside metabolism. In Caco-2 monolayers, trans-piceid displayed measurable bidirectional permeability in the 10^−6^ cm/s range, but the apparent permeability decreased during six hours of exposure as resveratrol and glucuronide metabolites appeared [13]. Experiments with rat intestinal fractions implicated membrane-associated lactase-phlorizin hydrolase and cytosolic β-glucosidase in deglycosylation, with D-gluconolactone inhibiting both activities. The authors proposed two routes: luminal hydrolysis by lactase-phlorizin hydrolase followed by passive entry of resveratrol, and uptake of intact trans-piceid followed by cytosolic hydrolysis. Resveratrol was subsequently converted predominantly to resveratrol-3-O-β-D-glucuronide and, to a lesser extent, resveratrol-4′-O-β-D-glucuronide, with UGT1A1 implicated in this process [13]. Oral absorption therefore comprises several concurrent processes rather than transport of intact polydatin alone. Analyses of intestinal contents and urinary metabolites further identified resveratrol, dihydroresveratrol, sulfate conjugates, and glucuronide products after polydatin administration, supporting contributions from intestinal conversion and phase-II metabolism to its disposition [33,34].

Rat pharmacokinetic studies are consistent with extensive presystemic conversion and limited systemic availability. After gavage at 50, 100, or 300 mg/kg, polydatin, resveratrol, glucuronidated polydatin, and glucuronidated resveratrol were detected in plasma within 10 min; intestinal and hepatic perfusion experiments supported first-pass deglycosylation and glucuronidation [35]. In a separate LC-MS/MS study, the same range of oral doses produced substantially lower dose-normalized exposure than a 20 mg/kg intravenous reference, giving an absolute oral bioavailability of approximately 2.9% [14]. At an oral dose of 20 mg/kg, simultaneous quantification showed a Tmax of approximately 0.33 h for both polydatin and resveratrol and a polydatin terminal half-life of approximately 1 h. Rapid hydrolysis by rat fecal S9 fractions provided additional evidence for microbial contribution to resveratrol formation [15]. A subsequent disposition study found that trans-resveratrol-3-O-glucuronide reached higher concentrations than intact polydatin and resveratrol in plasma, urine, and bile and accounted for 52.8% of the administered dose recovered in urine over 72 h [36].

Human evidence is restricted to exploratory bioanalysis. In six volunteers receiving commercial tablets containing 40 or 75 mg polydatin, plasma polydatin was quantifiable in only two samples, at 10.06 and 65.07 nM, and urinary polydatin was detected in one participant after repeated intake [16]. Sparse sampling and the lower analytical sensitivity for resveratrol prevent this study from defining human pharmacokinetics or the complete metabolite profile, but the observations are consistent with low and variable circulating concentrations of polydatin.

Current evidence therefore characterizes orally administered polydatin by physical-form-dependent behavior, measurable intestinal permeability, extensive intestinal and hepatic conversion, and low systemic availability. Plasma exposure has been examined more thoroughly than tissue-specific distribution, while sulfate and glucuronide profiles in target organs remain insufficiently defined. Further clarification of these exposure relationships will strengthen the mechanistic interpretation of polydatin studies and provide a pharmacokinetic basis for evaluating formulation-dependent changes in bioavailability and tissue distribution.

## 3. Pharmacological Mechanisms of Polydatin

**Figure 3:**
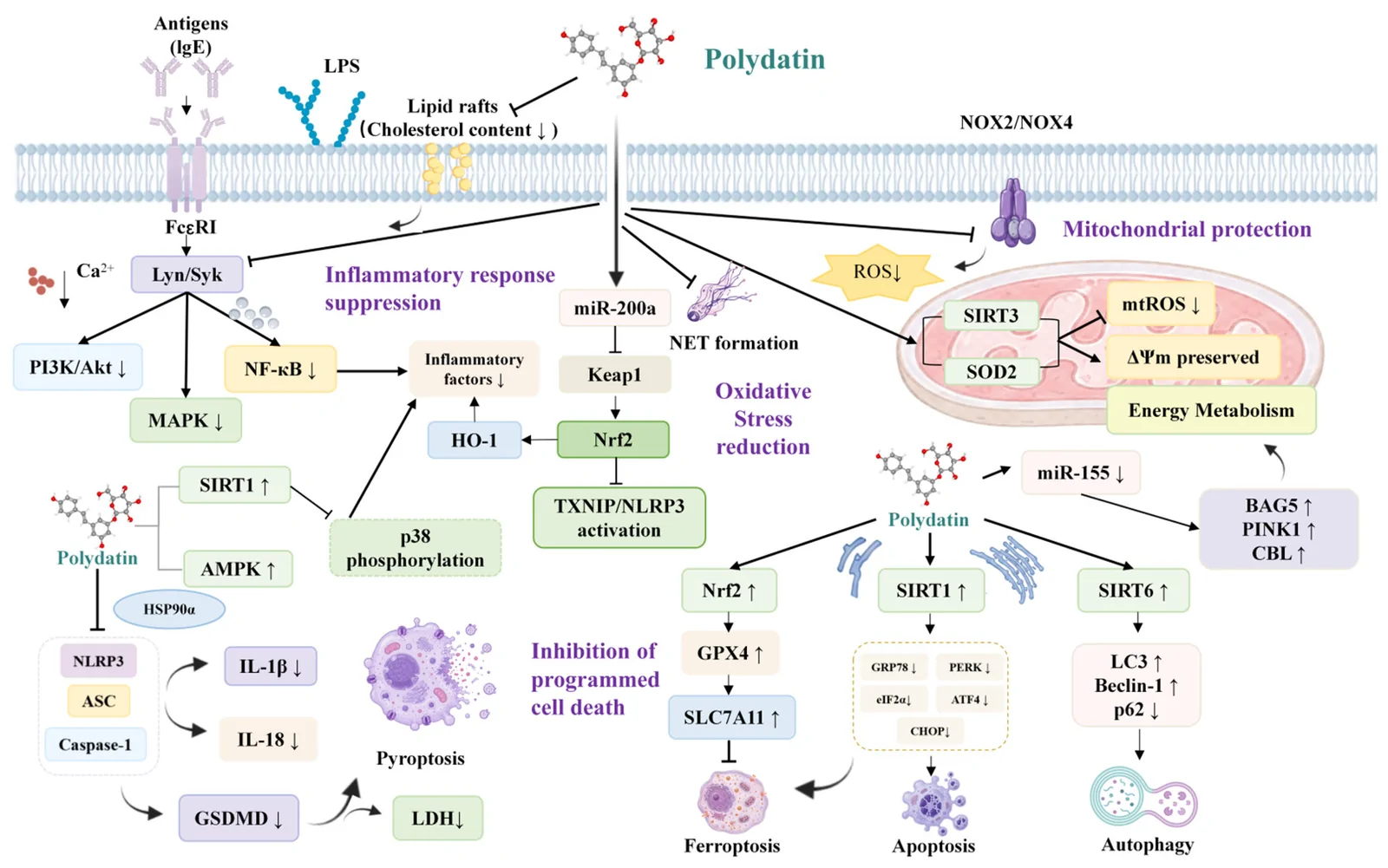
Reported pharmacological mechanisms of polydatin. Polydatin regulates redox homeostasis, inflammatory signaling, mitochondrial function, organelle quality control, and regulated cell death. Reported mechanisms include inhibition of FcεRI-associated Lyn/Syk, MAPK, PI3K/Akt, and NF-κB signaling; disruption of lipid-raft-associated inflammatory signaling; suppression of NOX2/NOX4-dependent reactive oxygen species production and neutrophil extracellular trap formation; and regulation of the miR-200a/Keap1/Nrf2, SIRT1/p38, and SIRT3/SOD2 pathways. Polydatin also disrupts HSP90α-dependent NLRP3 complex stability and reduces caspase-1/GSDMD-associated pyroptosis. Nrf2-, GPX4-, and SLC7A11-related responses contribute to ferroptosis suppression, whereas SIRT1-associated inhibition of endoplasmic reticulum stress, SIRT6-dependent autophagy, and miR-155/BAG5/PINK1/CBL-related mitophagy contribute to organelle homeostasis. The mechanisms shown were reported in different experimental models and represent parallel pharmacological actions of polydatin. Arrows indicate activation or positive regulation, whereas blunt-ended lines indicate inhibition. Upward (↑) and downward (↓) arrows denote increases and decreases, respectively. Colors are used for visual organization only and do not indicate evidence strength. Abbreviations: Akt, protein kinase B; AMPK, AMP-activated protein kinase; ASC, apoptosis-associated speck-like protein containing a CARD; ATF4, activating transcription factor 4; BAG5, BCL2-associated athanogene 5; CBL, Cbl proto-oncogene; CHOP, C/EBP homologous protein; eIF2α, eukaryotic translation initiation factor 2α; FcεRI, high-affinity IgE receptor; GPX4, glutathione peroxidase 4; GRP78, glucose-regulated protein 78; HO-1, heme oxygenase 1; HSP90α, heat shock protein 90α; IL, interleukin; Keap1, Kelch-like ECH-associated protein 1; LC3, microtubule-associated protein 1 light chain 3; LDH, lactate dehydrogenase; LPS, lipopolysaccharide; MAPK, mitogen-activated protein kinase; miR, microRNA; mPTP, mitochondrial permeability transition pore; mtROS, mitochondrial reactive oxygen species; NET, neutrophil extracellular trap; NF-κB, nuclear factor-κB; NLRP3, NLR family pyrin domain containing 3; NOX, NADPH oxidase; Nrf2, nuclear factor erythroid 2-related factor 2; PERK, protein kinase R-like endoplasmic reticulum kinase; PI3K, phosphatidylinositol 3-kinase; PINK1, PTEN-induced kinase 1; SLC7A11, solute carrier family 7 member 11; SOD2, superoxide dismutase 2; TXNIP, thioredoxin-interacting protein; ΔΨm, mitochondrial membrane potential.

Mechanistic studies link polydatin to redox regulation, inflammatory signaling, organelle homeostasis, and regulated cell death [8]. Most evidence comes from cells and animal models treated with free polydatin. Target-binding assays, pathway inhibitors, and genetic interventions provide stronger mechanistic support than concurrent changes in signaling proteins and disease-related endpoints (Table 1, Figure 3). Table 1 summarizes the mechanistic categories most relevant to delivery design; the complete study-level evidence matrix is provided in Supplementary Table S5.

### 3.1. Redox Regulation and Mitochondrial Function

Redox regulation is consistently reported for polydatin. In cell-free micellar and liposomal systems, piceid inhibited linoleic acid peroxidation, indicating protection against membrane lipid peroxidation in addition to solution-phase radical scavenging [37]. In cultured cardiomyocytes and rats exposed to angiotensin II (Ang II), polydatin reduced NADPH oxidase activity, NOX2/NOX4 expression, and superoxide generation, changes accompanied by less hypertrophic remodeling [38]. Similar coupling of redox, inflammatory, and apoptotic responses was observed in D-galactose-induced liver and brain injury [39].

Polydatin may also limit oxidative injury by preserving mitochondrial integrity. In rats subjected to hemorrhagic shock, polydatin preserved mitochondrial membrane potential in arterial smooth muscle cells, limited mitochondrial permeability-transition-pore opening and swelling, and reduced lysosomal destabilization [40]. In IL-1β-stimulated chondrocytes and osteoarthritic joints, polydatin increased SIRT3 and SOD2 expression, lowered mitochondrial superoxide production, and improved mitochondrial membrane potential, supporting SIRT3/SOD2-associated mitochondrial regulation [41].

The Keap1/Nrf2 pathway connects redox regulation with inflammatory signaling. In fructose-exposed hepatic cells and rats, polydatin increased microRNA-200a, reduced Keap1 expression, enhanced Nrf2-dependent antioxidant responses, and suppressed ROS-sensitive TXNIP/NLRP3 activation [42]. A microRNA-200a mimic and Keap1 or TXNIP silencing linked Keap1 suppression and Nrf2 activation to reduced oxidative stress and inflammasome signaling. Collectively, these studies describe polydatin as a regulator of redox-sensitive signaling and organelle function rather than a nonspecific antioxidant.

### 3.2. Inflammatory and Innate Immune Signaling

Polydatin acts at several levels of inflammatory signaling. In mast cells, IgE/antigen-mediated FcεRI crosslinking activates Lyn and Syk, followed by MAPK, PI3K/Akt, NF-κB, calcium mobilization, and mediator release. Polydatin reduced Lyn and Syk activity in cell-free kinase assays, attenuated downstream signaling, and increased Nrf2/HO-1 signaling, with corresponding reductions in degranulation and cytokine production [43]. The kinase assays place the effect of polydatin at receptor-proximal signaling in addition to changes in downstream cytokine production.

Membrane organization and sirtuin signaling provide additional regulatory sites. In LPS-stimulated BV2 microglia, polydatin decreased cholesterol in isolated lipid-raft fractions and reduced lipid-raft formation, coupled to lower PI3K/Akt/NF-κB activation and inflammatory mediator production [44]. In sepsis-associated encephalopathy, polydatin increased SIRT1 activity and reduced p38 phosphorylation, mitochondrial dysfunction, ROS generation, and inflammatory cytokines. The SIRT1 inhibitor Ex527 attenuated these effects, whereas p38 inhibition reproduced part of the response, supporting the involvement of SIRT1-regulated p38 signaling [45].

Polydatin also modifies immune-cell effector functions. In lupus-prone mice and neutrophils from patients with systemic lupus erythematosus, polydatin reduced ROS production, neutrophil extracellular trap formation, and associated autoimmune pathology [46]. In crystal-induced inflammatory models, polydatin reduced IL-1β-related responses in THP-1 cells and was associated with PPAR-γ induction, lower NLRP3 signaling, and changes in ferritin-related iron handling in monosodium urate-induced arthritis [47,48]. In osteoarthritis models, polydatin also modified macrophage polarization and bone-remodeling responses [49]. No single inflammatory target is shared across these models. Polydatin instead affects receptor-proximal kinases, lipid-raft-associated signaling, redox-sensitive transcriptional pathways, and immune-cell effector responses in a cell- and stimulus-dependent manner.

### 3.3. Inflammasome Signaling and Regulated Cell Death

NLRP3 inflammasome activation integrates ionic disturbance, organelle stress, lysosomal injury, and endogenous damage signals. Following priming and activation, assembly of the NLRP3-ASC-caspase-1 complex promotes IL-1β and IL-18 maturation and gasdermin D-mediated pyroptosis [50]. Polydatin has been reported to affect inflammasome priming, complex stability, and pyroptotic execution.

In potassium oxonate-induced hyperuricemia and renal inflammation, polydatin reduced IκBα degradation, NF-κB p65 activation, and NLRP3/ASC/caspase-1 signaling while increasing AMPK phosphorylation and SIRT1 expression [51]. In dry eye models, reduced ROS generation and NF-κB nuclear translocation were accompanied by lower NLRP3 expression and caspase-1 activation in corneal epithelial cells [52]. Polydatin also reduced NLRP3/IL-1β/NF-κB signaling, mesothelial inflammatory responses, and fibrotic changes in high-glucose-induced peritoneal fibrosis [53]. These studies support inhibition of redox- and NF-κB-related inflammasome priming, although neither identifies a direct molecular target shared across models.

More specific target evidence has been reported for HSP90α. Lip-SMap protein profiling and cellular thermal shift assays identified HSP90α as a polydatin-binding protein. Microscale thermophoresis and isothermal titration calorimetry yielded dissociation constants of 1.37 and 1.34 μM, respectively, and polydatin inhibited HSP90α ATPase activity with an IC50 of approximately 2.73 μM. HSP90α deletion abolished further suppression of IL-1β secretion by polydatin, while co-immunoprecipitation and imaging experiments showed disruption of the HSP90α-NLRP3 interaction and loss of resting and activated NLRP3 complexes [11]. The combined binding, target-engagement, and genetic evidence supports HSP90α as a direct molecular target in this experimental setting. In gouty nephropathy, polydatin also reduced caspase-1 activation, GSDMD and GSDMD-N expression, IL-1β release, LDH release, and renal tubular epithelial-cell death, linking inflammasome inhibition with reduced pyroptotic membrane permeabilization [54].

Ferroptosis is an iron-dependent form of regulated cell death driven by membrane lipid peroxidation, with GPX4-mediated lipid hydroperoxide reduction serving as a central defense mechanism [55]. In hemin-treated Neuro2A cells and a traumatic brain injury model, polydatin reduced iron accumulation and membrane lipid peroxidation and restored selenium-dependent glutathione peroxidase activity, accompanied by changes in GPX4- and SLC7A11-related responses [56]. In pancreatic β-cells exposed to palmitate under high-glucose conditions, molecular docking and a GPX4 promoter-luciferase reporter screen identified polydatin as a candidate regulator of GPX4 expression. Polydatin increased GPX4, SLC7A11, and Nrf2-related responses and reduced ferroptosis-associated changes [12]. The reporter system measured GPX4 promoter regulation rather than direct GPX4 enzymatic activation; these findings therefore support regulation of the Nrf2/GPX4 defense system rather than direct binding to GPX4. Overall, polydatin affects inflammasome-dependent pyroptosis and ferroptosis through distinct molecular processes.

### 3.4. Autophagy and Mitophagy

Polydatin has been reported to regulate autophagy and selective mitochondrial clearance in a context-dependent manner; functional perturbation, rather than marker changes alone, is needed to relate these processes to tissue protection [57,58]. In septic myocardial injury, polydatin increased SIRT6 expression and produced an autophagy-associated profile characterized by increased LC3 processing and Beclin-1 and reduced p62. SIRT6 silencing attenuated these marker changes, and 3-methyladenine weakened the reductions in apoptosis and inflammatory cytokines, supporting a functional contribution of SIRT6-dependent autophagy [59]. In atherosclerotic mice, pharmacological inhibition or induction of autophagy respectively weakened or reinforced the response to polydatin, linking PI3K/Akt/mTOR inhibition and autophagy restoration to reduced plaque formation [60]. Mitophagy-related evidence includes human trisomy 21 fibroblasts, in which polydatin reduced microRNA-155, restored BAG5, PINK1, and CBL expression, increased mitochondrial-lysosomal colocalization, and improved mitochondrial bioenergetics [61]. A later study in fatty-acid-exposed HepG2 cells and high-fat-diet rats linked the effects of free polydatin on mitochondrial dynamics and PINK1/Parkin-associated mitophagy to BMAL1; BMAL1 knockdown abolished the reported metabolic and mitochondrial responses [62]. Both studies used free polydatin and therefore provide mechanistic endpoints rather than delivery-system evidence.

### 3.5. Implications for Delivery Design

Several mechanistic features of polydatin impose specific requirements on delivery (Table 1). Nrf2/Keap1, HSP90α/NLRP3, GPX4, sirtuins, and the autophagy machinery are intracellular, while mitochondrial and lysosomal effects require access to particular subcellular environments [11,42,59]. The relevant cell population also varies among disease settings, including inflammatory cells, vascular and epithelial cells, chondrocytes, and neurons. Tissue accumulation alone does not establish that polydatin has reached the cells or intracellular sites required for pathway modulation [63,64]. The available studies do not define a common concentration threshold or exposure duration. Acute injury and chronic inflammatory models use different schedules, but few compare rapid with sustained exposure. Cell studies relate nominal extracellular concentrations to pathway changes without measuring intracellular polydatin or concentrations required for target engagement. Delivery studies could address these gaps by linking drug release, cellular uptake, and intracellular exposure to pathway-specific endpoints and to the magnitude and duration of local exposure.

## 4. Delivery Strategies for Polydatin

**Figure 4:**
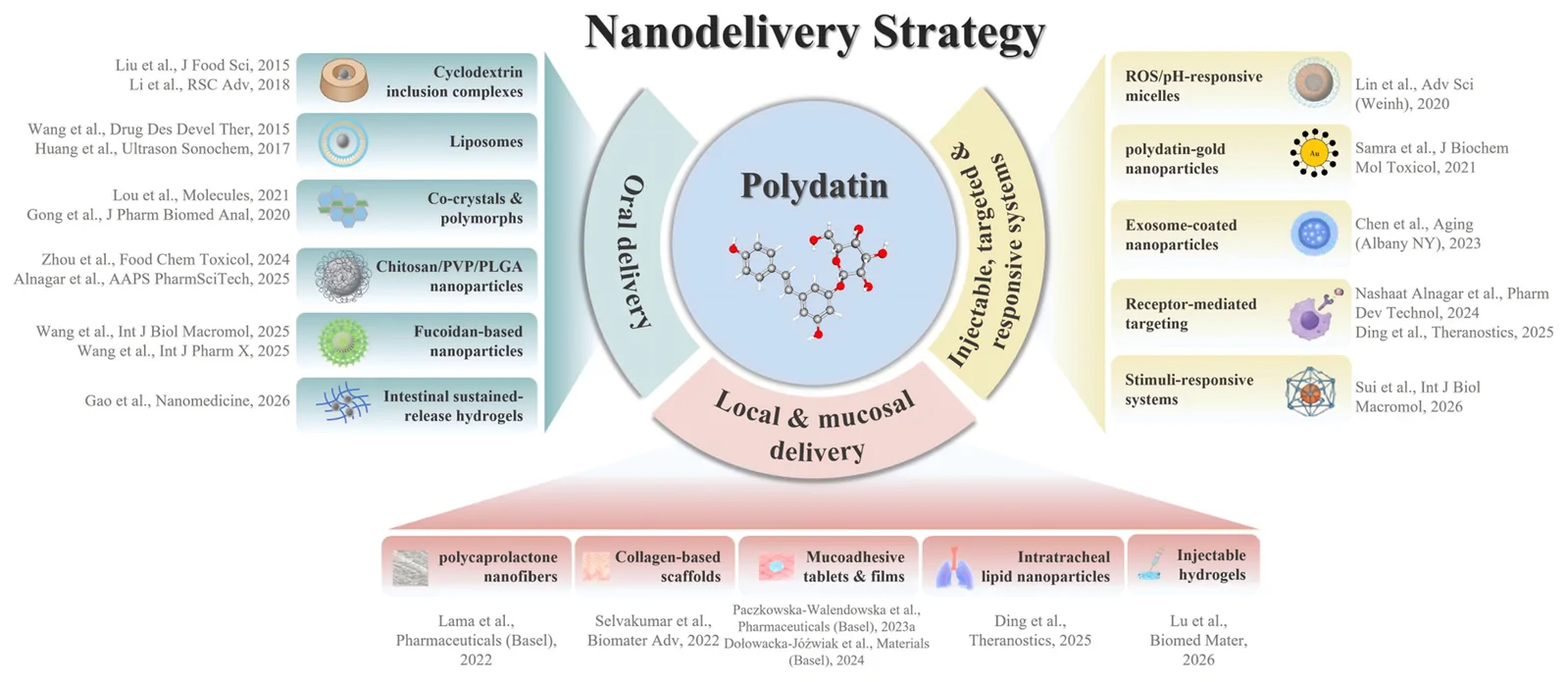
Route- and function-based classification of polydatin formulation and delivery strategies. Representative systems are organized according to their principal administration route and delivery function rather than carrier material alone. Oral strategies include cyclodextrin inclusion complexes, solid-state modifications, liposomes, polymeric or polysaccharide nanoparticles, and intestinal sustained-release hydrogels, which have been developed to improve solubility, dissolution, gastrointestinal release, epithelial uptake, or systemic exposure. Local and mucosal systems include polycaprolactone nanofibers, collagen-based scaffolds, mucoadhesive tablets & films, intratracheally administered lipid nanoparticles, and hydrogels designed to prolong residence and regulate release at the application site. Injectable, targeted, and responsive platforms comprise ROS/pH-responsive micelles, polydatin-gold nanoparticles, exosome-coated nanoparticles, ligand-modified carriers, and pathological stimulus-responsive systems intended to modify tissue or cellular distribution and control drug release. These categories are not mutually exclusive because individual formulations may combine route-specific delivery with targeting, stimulus responsiveness, or co-delivery. Cyclodextrin complexes, co-crystals, and polymorphs are non-nanoscale formulation-enabling strategies, whereas some mucosal preparations contain multicomponent botanical extracts rather than purified polydatin. PCL, polycaprolactone; PLGA, poly(lactic-co-glycolic acid); PVP, polyvinylpyrrolidone; ROS, reactive oxygen species [22,23,25,28,29,65,66,67,68,69,70,71,72,73,74,75,76,77,78,79,80,81].

Because the same carrier can perform differently after oral, intravenous, intraperitoneal, or local administration, this section is organized primarily by administration route rather than material class. Evidence is considered at three levels: formulation properties, including solubility, stability, and release; delivery performance, including pharmacokinetics, tissue distribution, and cellular uptake; and biological responses in experimental models. Improvement at one level does not establish improvement at the others (Figure 4).

### 4.1. Oral Delivery

Oral formulations pursue two distinct goals: increasing systemic exposure or prolonging intestinal residence. Non-nano strategies provide important comparators. Cyclodextrin inclusion complexes [29,65] and an L-proline co-crystal [66] improved in vitro solubility or stability but were not linked to oral pharmacokinetics. By contrast, a trihydrate polymorph produced faster absorption and approximately 1.5-fold greater exposure than the anhydrous form after a matched oral dose [28]. Among nanoscale systems, oral liposomes provided the first direct pharmacokinetic comparison with a dose-matched polydatin suspension [67]. The formulation increased AUC 2.83-fold and prolonged apparent half-life and mean residence time, whereas the 1.21-fold increase in Cmax was not reported as significant. A separate ultrasound-assisted liposome study optimized encapsulation and processing but did not evaluate pharmacokinetics or in vivo delivery [68].

Polymeric and polysaccharide carriers have used chitosan, PVP, and PLGA to improve wetting, mucoadhesion, or release control [82,83]. Dose-matched formulations generally produced stronger pharmacodynamic responses than free polydatin in diabetes [21], associated nephropathy and liver injury [84,85], and oxaliplatin-induced intestinal injury or developing pulmonary fibrosis [69,70]. However, these studies did not measure pharmacokinetics, mucus residence, tight-junction modulation, or epithelial transport, so the basis of the improved oral responses remains unresolved.

Two sequential studies from the same group evaluated fucoidan-based oral nanoparticles in cisplatin-induced kidney injury. The first used a fucoidan/carboxymethyl-chitosan carrier and reported a 2.33-fold increase in AUC together with higher renal polydatin concentrations than free drug [71]. The later formulation incorporated the pharmacologically active chemical chaperone 4-PBA and reported an approximately twofold AUC increase together with P-selectin-associated uptake and endoplasmic reticulum localization experiments [72]. Because both the carrier and 4-PBA may contribute to the response, the later report is best interpreted as an extension of the earlier platform rather than independent replication. Across the three direct oral comparisons, formulated polydatin increased AUC by approximately 2.0–2.83-fold, whereas changes in Cmax ranged from 1.21- to 3.77-fold. The two fucoidan studies were small (*n* = 3 per group) and contained internal inconsistencies in the reported AUC units; Table 2 therefore reproduces the source values. Collectively, these data support increased systemic exposure but not selective kidney delivery, and they do not resolve intestinal conversion or metabolite disposition.

Other oral systems are intended to increase intestinal exposure rather than systemic absorption. PVP-polydatin nanoparticles improved outcomes in oxaliplatin-induced intestinal injury, but intestinal polydatin concentrations were not measured [70]. A graphene oxide-polyacrylic acid hydrogel was designed to swell after oral administration and sustain polydatin release during radiation injury [73]. For these systems, gastrointestinal residence and polydatin concentrations in mucus, epithelium, and deeper tissue are more informative than plasma exposure alone. Table 2 and Table 3 summarize direct PK and broader oral evidence.

### 4.2. Intravenous and Intraperitoneal Delivery

The existing injectable studies used either intravenous or intraperitoneal administration. Both avoid gastrointestinal processing, but the delivery questions are not identical. Intravenous carriers require evaluation of interactions with blood components, mononuclear phagocyte system clearance, vascular transport, tissue distribution, and cellular internalization [17,18]. Intraperitoneal administration also avoids intestinal absorption but should not be treated as pharmacokinetically equivalent to intravenous injection (Table 4).

The principal intravenous study used ROS- and pH-responsive micelles in carbon tetrachloride-induced liver fibrosis. After 3 weeks of model induction, mice received the micelles by tail-vein injection every other day for a further 3 weeks. Each dose contained 40 mg/kg carrier and 2.5 mg/kg polydatin; blank micelles were given intravenously at 40 mg/kg, whereas free polydatin was administered intraperitoneally at 2.5 mg/kg [23]. The micelles accumulated in the liver, with early uptake attributed largely to the hepatic mononuclear phagocyte system. Fluorescence imaging indicated greater probe release in fibrotic liver, but measured organ polydatin concentrations did not differ between healthy and fibrotic mice. The route mismatch between micellar and free polydatin groups limits direct comparison of efficacy.

Other injectable studies used intraperitoneal administration. Exosome-coated polydatin nanoparticles and route-matched control preparations were injected immediately after abdominal irradiation; the methods reported 20 mg/kg polydatin and 2 mg/kg exosomes [22]. The formulation increased survival and altered intestinal histology and mitochondrial functional measurements, but comparative plasma or tissue concentration-time data were not reported. The study therefore supports biological activity after exosome-associated delivery but does not establish increased bioavailability. Polydatin-associated gold nanoparticles were administered intraperitoneally for 21 days in tumor-bearing mice, with a 20 mg/kg free-polydatin group and blank gold nanoparticle controls [74]. Although the route-matched controls aid interpretation, incomplete reporting of polydatin loading, release, and pharmacokinetics limits assessment of the carrier contribution. Injectable administration has therefore been shown to be feasible, but current evidence does not establish a consistent improvement in the systemic disposition or tissue delivery of polydatin.

### 4.3. Local and Mucosal Delivery

Local delivery shifts the evaluation focus from plasma exposure to residence at the application site, tissue penetration, release duration, material degradation, and local tolerance [19,20]. Polydatin-loaded polycaprolactone nanofibers released polydatin for up to 14 days without a marked initial burst and supported osteogenic differentiation in vitro [77]. No implantation study was performed, so residence, degradation, and local polydatin exposure remain to be established in vivo.

A collagen-oxidized pullulan scaffold released approximately 63% of its polydatin within 48 h and more than 90% within 96 h. When applied to excisional wounds, the polydatin-containing scaffold was compared with untreated wounds, collagen alone, and collagen-oxidized pullulan controls and was followed for up to 16 days in normal rats and 21 days in diabetic rats [25]. The matrix increased wound closure and altered tissue-repair markers, but local polydatin concentrations and scaffold degradation were not quantified. A more recent oxidized hyaluronic acid/catechol-chitosan hydrogel contained 0.4 mg/mL polydatin and 0.068 mg/mL tetramethylpyrazine and released approximately 80% of both agents within 48–52 h [78]. Wounds were followed for 14 days, but the in vivo comparison included the blank hydrogel and the dual-drug hydrogel without single-drug hydrogel groups; the individual contribution of polydatin cannot be determined. A carrier-free hydrogel co-assembled from mangiferin and polydatin has been reported for MRSA-infected diabetic wounds, with demonstrated sustained release, antibacterial and anti-biofilm effects, macrophage polarization, revascularization, and improved wound repair in rats. However, the absence of component-specific controls means these outcomes can only be attributed to the complete non-nano binary hydrogel, not to polydatin alone [91]. These local delivery systems and their principal evidence boundaries are summarized in Table 5.

Direct mucosal delivery has been explored using formulations placed on oral surfaces. Extract-containing electrospun nanofibers improved the solubility and mucosal permeation of polydatin and resveratrol [92], whereas mucoadhesive tablets prolonged local release and mucosal residence [79], and perio-dontal films provided sustained local release [80]. A separate study used hot-melt extrusion to prepare solid dispersions of polydatin-rich *Polygoni cuspidati* extract with polyvinyl polymers and incorporated selected extrudates into mucoadhesive buccal tablets; the resulting formulation increased polydatin release and showed in vitro mucoadhesive performance [93]. Because these preparations contain multicomponent botanical extracts, their release and biological effects cannot be assigned to polydatin alone (Table 6).

### 4.4. Targeted and Responsive Systems

Targeted and responsive systems seek to control complementary stages of polydatin delivery: targeted carriers alter tissue or cellular distribution, whereas responsive materials regulate drug release or carrier activity after exposure to a local stimulus. Evidence for targeting ranges from receptor-blocking experiments in cells to ligand-controlled distribution studies in vivo. In A549 cells, hyaluronic acid- and lactoferrin-coated polydatin/PLGA nanoparticles increased cellular uptake, and CD44 blockade reduced uptake of the hyaluronic acid-coated formulation [76]. The oral fucoidan/4-PBA system used P-selectin inhibition to examine carrier uptake and fluorescence colocalization to assess endoplasmic reticulum localization [72]. The intratracheally administered SPFPT-modified lipid nanoparticle included both ligand and component controls when delivering polydatin and siRNA targeting importin 7 to mechanically activated alveolar epithelial cells [75]. Importin 7 silencing reduced p38 nuclear translocation, whereas polydatin contributed to NF-κB inhibition. Cy5-labeled formulations containing 5 μg siRNA were assessed 2 h after administration against untargeted and scrambled-peptide controls, and the combined formulation reduced TNF-α, IL-6, and HMGB1 more than the polydatin-only or siRNA-only nanoparticles. These experiments associate specific ligands with carrier uptake or accumulation, although the receptor for SPFPT remains unidentified. Because fluorescence does not directly measure drug delivery, quantitative imaging combined with polydatin measurement would distinguish carrier biodistribution from target-cell drug exposure [94].

Taken together, these examples illustrate three evidence levels that should not be conflated. Fluorescence-only findings indicate carrier or surrogate distribution, whereas CD44 blockade in A549 cells and SPFPT negative-control experiments support mechanism-based carrier targeting; however, the tracked analytes were RhB and Cy5-siRNA rather than polydatin [75,76]. The fucoidan systems measured tissue polydatin, but only at a single time point and across multiple organs. Moreover, mouse DiR renal fluorescence was not concordant with rat tissue-polydatin concentrations, which were highest in the liver for Fu-4-PBA/polydatin nanoparticles [71,72]. These limitations preclude a drug-level active kidney-targeting claim, and none of the current studies combines quantitative tissue polydatin with a matched non-targeted comparator and mechanistic evidence. This distinction provides the context for evaluating stimulus-responsive release separately from selective drug targeting.

After a carrier reaches the intended tissue or cell population, stimulus-responsive design can regulate polydatin release or activate a functional carrier. Intravenous micelles released 9.38% of polydatin over 24 h at pH 7.4 and 89.27% under combined pH 5.0 and oxidative conditions [23]. Because pH 5.0 is more representative of endosomal or lysosomal compartments than the extracellular environment of fibrotic liver [95], this profile is more consistent with activation after cellular uptake. Two ZIF-8 systems illustrate a further distinction. A Cu-doped ZIF-8 nanozyme hydrogel combined pH-responsive carrier activity with local antibacterial and wound-healing effects [81], whereas chitosan-coated polydatin@ZIF-8 nanoparticles showed 91.34% loading efficiency, pH-dependent release, and in vitro antibacterial activity without an in vivo exposure, efficacy, or selective-targeting experiment [90]. These systems demonstrate responsive release or carrier activity, but neither acidic release nor fluorescence alone establishes selective delivery of polydatin (Table 4 and Table 5).

## 5. Polydatin Delivery Systems in Disease Models

Formulation characterization, release studies, pharmacokinetics, and cellular uptake describe delivery performance, whereas disease models allow the pharmacological relevance of a formulation to be assessed. In these models, the key question is whether altered release, exposure, or distribution changes the magnitude or duration of the pharmacological effects of polydatin in affected tissues and cells. Effects contributed by carrier materials or co-delivered agents must also be distinguished from those attributable to polydatin. Polydatin delivery systems have been examined in models of diabetes and its complications [21], kidney and intestinal injury [70,71], and pulmonary and hepatic fibrosis [23,69]. Cancer and tissue-repair applications have also been explored [24,25]. The following subsections examine the pharmacological effects reported in these models and the evidence linking them to polydatin delivery (Table 7).

### 5.1. Diabetes and Its Complications

Oral chitosan nanoparticles have been examined in a closely related experimental series using nicotinamide/streptozotocin-induced type 2 diabetic rats. In the systemic metabolic study, polydatin-loaded chitosan nanoparticles and free polydatin were administered by oral gavage at 50 mg/kg once daily for 28 days. Compared with dose-matched free polydatin, the nanoparticle formulation produced larger reductions in fasting glucose, HbA1c, and insulin resistance, increased hepatic glycogen, and improved the serum lipid profile [21]. Studies using a similar formulation, dose, and four-week schedule extended these findings to early diabetic nephropathy and diabetic liver injury. The nephropathy study reported greater improvements in renal function indices, oxidative stress, NF-κB/COX-2 signaling, and circulating cytokines [85], whereas the liver study showed improved hepatic glucose metabolism and antioxidant status, lower transaminase activities, and modulation of GLUT2, glucokinase, TNF-α, and IL-1β [84]. The consistency of these metabolic, renal, and hepatic responses supports greater oral pharmacodynamic activity of the chitosan formulation than free polydatin, although the exposure changes responsible for this difference were not measured.

Local delivery has extended polydatin application to diabetic wounds and ulcers. In diabetic excision wounds, complete closure occurred by day 21 with a polydatin-containing collagen-oxidized pullulan scaffold, compared with day 27 for the unloaded scaffold and day 30 in untreated animals [25]. An oxidized hyaluronic acid/catechol-chitosan hydrogel co-delivering tetramethylpyrazine and polydatin produced nearly complete closure by day 14 and altered markers of angiogenesis and matrix remodeling [78]. An infection-responsive hydrogel containing polydatin-loaded Cu-doped ZIF-8 nanozymes reduced bacterial burden and accelerated closure of diabetic oral ulcers [81]. The scaffold provided sustained local polydatin delivery, whereas the two hydrogels combined polydatin with complementary proangiogenic or antibacterial components. The effects of the latter formulations therefore reflect the complete delivery systems rather than polydatin alone.

Diabetic vascular and myocardial injury represents a further setting for polydatin delivery. Under high-glucose conditions, free polydatin restored endothelium-dependent relaxation through PPARβ/NO signaling and preserved endothelial mitochondrial membrane potential and Drp1-associated mitochondrial dynamics [96,97]. In diabetic myocardial models, Caveolin-1 deletion or knockdown attenuated the response to polydatin, while a separate vascular study reported lower NLRP3 and VCAM-1 expression after polydatin treatment [98]. These studies provide defined pharmacodynamic endpoints for evaluating polydatin delivery in diabetic vascular and myocardial injury.

### 5.2. Kidney Injury

Two sequential studies from the same group linked oral pharmacokinetics and organ distribution with renal outcomes in cisplatin nephrotoxicity. The first used self-assembled fucoidan/carboxymethyl-chitosan nanoparticles, which increased AUC approximately 2.3-fold and increased renal polydatin concentrations relative to free polydatin. The formulation also produced larger reductions in renal dysfunction, tubular injury, oxidative stress, DNA damage, cytosolic mitochondrial DNA, and cGAS-STING-related inflammation [71]. The later system incorporated 4-PBA into the fucoidan carrier and approximately doubled AUC while adding P-selectin-associated uptake and endoplasmic reticulum localization experiments. It also produced stronger renal and molecular responses than free polydatin [72]. However, 4-PBA is an active chemical chaperone, so these effects reflect both altered polydatin exposure and the pharmacological contribution of the co-component. Both studies began treatment before cisplatin administration and should therefore be interpreted as prevention or attenuation of developing injury. Their shared investigators, model, and endpoints also mean that the second study extends, rather than independently replicates, the first. Higher renal concentrations occurred alongside substantial hepatic distribution, supporting enhanced renal exposure but not kidney-selective delivery. Studies of free polydatin have additionally identified NF-κB inhibition and Nrf2/HO-1 activation, SIRT6-dependent autophagy, and reduced YAP expression or nuclear translocation as renal pharmacodynamic endpoints [99,100,101].

### 5.3. Intestinal Injury

Intestinal studies have examined systemic intervention after radiation injury, oral nanoparticle delivery during chemotherapy, and sustained luminal release. In the radiation-injury model, exosome-coated polydatin nanoparticles administered immediately after irradiation improved survival and leukocyte recovery and reduced diarrhea, villus loss, crypt injury, γ-H2AX, inflammatory mediators, mitochondrial dysfunction, and cellular senescence [22]. These findings indicate that post-injury administration supported both systemic recovery and intestinal epithelial preservation. In an oxaliplatin injury model, mice received free polydatin or polyvinylpyrrolidone-polydatin nanoparticles orally at 50 or 100 mg/kg for seven days, with oxaliplatin administered on days 3–6. At 100 mg/kg, the nanoparticle group showed greater preservation of body weight, colon length, crypt architecture, ileal villus morphology, goblet cells, and mucosal integrity than dose-matched free polydatin. Lower ROS, γ-H2AX, cGAS, STING, IL-1β, and TNF-α and improved mitochondrial indices linked the stronger tissue response to attenuation of epithelial stress and inflammation [70]. An orally administered graphene oxide-polyacrylic acid hydrogel was also reported to sustain intestinal polydatin release and improve epithelial and hematological endpoints after irradiation [73]. Free polydatin has separately been reported to preserve epithelial junction proteins and reduce Nrf2/SLC7A11/GPX4-related ferroptotic injury in DSS colitis [102], providing an additional mechanism that could be examined in future intestinal delivery studies. Collectively, systemic rescue, oral nanoparticle delivery, and sustained luminal release have each extended the intestinal effects of polydatin across radiation-, chemotherapy-, and inflammation-related injury models.

### 5.4. Pulmonary Injury and Fibrosis

Pulmonary delivery studies address two route-specific objectives: local intervention after acute mechanical injury and repeated oral administration during fibrosis development. In ventilator-induced lung injury, SPFPT-modified lipid nanoparticles co-delivering polydatin and siRNA targeting importin 7 were administered intratracheally after 4 h of mechanical ventilation. Compared with nanoparticles carrying either component alone, the combined formulation produced greater inhibition of p38 and NF-κB signaling, lowered TNF-α, IL-6, and HMGB1 in bronchoalveolar lavage fluid, and reduced pulmonary edema, neutrophil infiltration, and histological injury [75]. Cy5 imaging localized the siRNA-containing formulation to mechanically activated alveolar epithelial cells but did not measure the distribution of polydatin.

By contrast, an oral chitooligosaccharide-coated PLGA formulation was evaluated during the development of bleomycin-induced pulmonary fibrosis. The formulation was administered once daily at a dose equivalent to 25 mg/kg polydatin from day 1 to day 17; bleomycin was given 1 h after the third dose on day 3. Compared with dose-matched free polydatin, the nanoparticle group showed greater preservation of lung architecture and larger reductions in collagen deposition, α-SMA, TGF-β, lipid peroxidation, TNF-α, and IL-1β [69]. Because dosing preceded bleomycin exposure, the results demonstrate prevention or attenuation of developing fibrosis rather than intervention in established fibrosis.

Mechanistic studies of free polydatin extend the pulmonary evidence beyond these delivery models. After *Mycoplasma pneumoniae* infection, polydatin suppressed NLRP3/NF-κB signaling and caspase-1/GSDMD-related epithelial pyroptosis [103,104]. Other studies reported attenuation of methotrexate-induced pulmonary fibrosis and reduced S100B-dependent neutrophil extracellular trap formation in traumatic brain injury-associated lung injury [105,106]. These pathways provide defined pharmacodynamic endpoints for pulmonary delivery studies. Current formulation evidence therefore supports local co-delivery after acute mechanical injury and repeated oral administration during fibrosis development, with established pulmonary fibrosis representing a relevant setting for subsequent evaluation.

### 5.5. Liver Fibrosis

ROS- and pH-responsive micelles were evaluated in a carbon tetrachloride (CCl_4_)-induced liver fibrosis model using an intervention schedule initiated after fibrosis had begun to develop. Following three weeks of CCl_4_ exposure, the micelles were administered by tail-vein injection every other day for a further three weeks at 40 mg/kg carrier, equivalent to 2.5 mg/kg polydatin. The formulation reduced hepatocyte apoptosis, inflammatory macrophage activity, hepatic stellate cell activation, collagen deposition, and fibrosis-related markers [23]. Cellular and tissue experiments were consistent with uptake by hepatocytes, macrophages, and hepatic stellate cells and with release under ROS-rich and acidic conditions. The micellar system integrates microenvironment-responsive release with pharmacological effects in several cell populations involved in fibrosis. The unloaded micelles also reduced oxidative stress, indicating that ROS scavenging by the carrier complemented the activity of polydatin. Thus, the reported antifibrotic response reflects the combined contribution of responsive delivery, polydatin, and the carrier material. Direct comparison with free polydatin remains limited by the use of intravenous micelles and intraperitoneal free polydatin.

### 5.6. Cancer and Other Exploratory Applications

Cancer-related formulations have been examined in chemoprevention, combination treatment, and targeted cellular delivery. Oral PLGA-polydatin nanoparticles administered at 7.5–30 mg/kg three times weekly throughout a 14-week 7,12-dimethylbenz[a]anthracene (DMBA) protocol reduced tumor incidence, tumor burden, lipid peroxidation, and proliferation-related markers in buccal carcinogenesis [24]. However, the absence of free-polydatin and unloaded-PLGA groups prevents separation of the formulation effect. In Ehrlich ascites carcinoma-bearing mice, intraperitoneal polydatin-gold nanoparticles containing 20 mg/kg polydatin, administered alone or with doxorubicin for 21 days, improved tumor-related and biochemical endpoints [74]. Hyaluronic acid/lactoferrin-coated PLGA nanoparticles increased CD44-associated uptake and cytotoxicity in A549 lung cancer cells [76], providing in vitro support for receptor-associated cellular delivery. In HER2-overexpressing SKBR3 cells, polydatin-loaded chitosan nanocapsules at 200 and 400 μg/mL reduced proliferative capacity by approximately 40% and 60%, respectively, after 72 h and produced cytoskeletal and nuclear changes, whereas empty nanocapsules did not reduce viability [88]. Gelatin-coated polycaprolactone nanoparticles produced stronger antiproliferative responses than free polydatin in radioresistant A549 cells and three-dimensional spheroids, although proteomic and in silico analyses indicated broadly similar molecular responses [89].

Mechanistic studies provide additional endpoints for cancer-oriented delivery. Combination studies reported that polydatin enhanced cisplatin-associated responses in non-small-cell lung cancer through NOX5-dependent ROS generation and endoplasmic reticulum stress/JNK/p38 signaling. During sunitinib treatment, polydatin reduced oxidative and inflammatory responses associated with NLRP3, MyD88, and NF-κB signaling. Separate work in head and neck squamous cell carcinoma linked polydatin to HDAC1 inhibition and reduced angiogenic activity [107,108,109]. Outside cancer, polymeric nanocapsules retained the anti-inflammatory and cytoprotective activity of polydatin in LPS-exposed hippocampal organotypic cultures [87], while amphiphilic chitosan nanocarriers improved behavioral, inflammatory, and oxidative endpoints in an aluminum chloride-induced Alzheimer-like rat model [86]. Polydatin-loaded polycaprolactone nanofibers promoted osteogenic differentiation in vitro [77], supporting their further evaluation in bone-repair models. Atherosclerosis represents another mechanism-based application because free polydatin has been reported to activate autophagy and suppress NLRP3 inflammasome-related pyroptosis in atherosclerotic lesions [110].

Across the disease models reviewed, dose-matched comparisons show that polydatin delivery systems produced larger pharmacodynamic responses than free polydatin in diabetes, renal and intestinal injury, and developing pulmonary fibrosis [21,69,70,71]. In cisplatin nephrotoxicity, two fucoidan studies combined plasma pharmacokinetics with single-time tissue measurements; stronger renal responses accompanied higher systemic exposure, but multi-organ enrichment did not establish kidney-selective delivery [71,72]. Local and responsive formulations prolonged release or residence and enabled stimulus-dependent material behavior [23,25], while multicomponent systems added antibacterial or combination functions [74,81]. Responses to the latter systems reflect the complete formulations rather than polydatin alone.

Several mechanisms established for free polydatin provide additional pharmacodynamic endpoints for delivery studies. These include HSP90α/NLRP3 regulation and pyroptosis [11,54], Nrf2/system Xc^−^/GPX4-dependent ferroptosis control [12,56,111], SIRT-dependent autophagy and mitophagy [10,59,61], and immune-cell processes involving neutrophil extracellular traps and lipid-raft-associated signaling [44,46]. Incorporating these intracellular endpoints alongside quantitative exposure measurements would clarify whether formulation-dependent changes in polydatin disposition modify target engagement and regulated cell-death pathways in relevant cell populations.

## 6. Safety, Pharmaceutical Development, and Regulatory Considerations

### 6.1. Safety Evidence and Remaining Risks

Current safety evidence consists mainly of short-term cytocompatibility, hemocompatibility, and selected animal observations. ROS- and pH-responsive micelles maintained more than 95% viability in hepatocytes, macrophages, and hepatic stellate cells at a polydatin concentration of 25 μg/mL [23]; a collagen-oxidized pullulan scaffold supported approximately 93% fibroblast viability and was also evaluated for hemolysis and erythrocyte adhesion [25]. Fucoidan/4-PBA nanoparticles produced a hemolysis rate of 4.92% at 10 μg/mL, and their cytocompatibility was examined in HK-2 cells [72]. In vivo, no overt histological injury was detected in the heart, liver, spleen, or lung after oral administration of fucoidan/carboxymethyl-chitosan nanoparticles at 100 mg/kg in the cisplatin nephrotoxicity study, while major-organ histology remained unremarkable 21 days after intratracheal administration of SPFPT-modified lipid nanoparticles [71,75]. These findings support only initial compatibility under the tested conditions; they do not establish repeat-dose safety, immune compatibility, or carrier clearance.

Accordingly, a lead polydatin formulation should undergo route- and duration-matched repeat-dose studies with toxicokinetic confirmation, recovery cohorts, clinical observations, hematology and coagulation testing, serum chemistry, urinalysis, organ weights, histopathology, and assessment of carrier or excipient persistence. Empty-carrier and free-polydatin comparators are essential, particularly for fucoidan-, chitosan-, exosome-, gold-, siRNA-, or 4-PBA-containing systems. Immunogenicity and immunotoxicity assessment should be product-specific: blood-contacting or inhaled particles require evaluation of complement activation, cytokine release, leukocyte and platelet effects, hemolysis, and hypersensitivity risk; proteinaceous, extracellular-vesicle, or surface-ligand components may additionally require anti-carrier antibody testing where biologically plausible. Local scaffolds require evaluation of degradation products, irritation, delayed inflammation, and tissue remodeling. Their omission from the included studies is an evidence gap, not evidence of safety [112].

### 6.2. Pharmaceutical Development and Critical Quality Attributes

Existing quality-control studies cover chemical identity, drug content, particle properties, stability, and route-relevant release. A validated fused-core HPLC method separated polydatin and resveratrol within 3 min and showed suitable precision and recovery for identity and content testing in commercial preparations [113]. Representative nanoliposomes had a mean particle size of 80.2 nm and an encapsulation efficiency of 88.4% [67], whereas oral chitosan nanoparticles measured 144.25 ± 3.37 nm, achieved an encapsulation efficiency of 96.74 ± 0.39%, and released less than 20% of polydatin within 12 h [21]. Fucoidan/4-PBA nanoparticles measured 102 ± 3.46 nm, had a zeta potential of −16.5 ± 0.49 mV and a drug-loading capacity of 10.34 ± 0.60%, and were monitored for particle-size and surface-charge stability over two months [72].

Release testing also reflected the intended formulation function: responsive micelles released 9.38% of polydatin over 24 h at pH 7.4 but 89.27% under combined acidic and oxidative conditions [23], whereas the collagen-oxidized pullulan scaffold released 62.6% within 48 h and more than 90% within 96 h [25]. These reports show that formulation-level measurements are feasible, but most are single-batch characterizations and do not establish manufacturing consistency or a product-level control strategy [27,114].

Consistent with quality-by-design principles, a development-ready control strategy for polydatin should link drug-substance and drug-product attributes to clinically relevant performance. Drug-substance testing should distinguish trans-polydatin from cis-polydatin and resveratrol and address assay, water content, solid form, source-related impurities, residual solvents, and degradants formed by light, oxidation, hydrolysis, or deglycosylation. Drug-product assays should distinguish total polydatin from encapsulated, surface-associated, and unencapsulated fractions. Mean particle size alone is insufficient; distribution width or polydispersity index, particle concentration, morphology, surface charge, ligand density, carrier molecular weight, loading, in vitro release, pH, osmolality, and residual solvents or crosslinkers should be measured as relevant. Sterility, endotoxin, and container-closure integrity are route-dependent release attributes. Independent batches, predefined acceptance criteria, and stability-indicating studies are needed to quantify batch-to-batch variability and detect aggregation, leakage, charge drift, release changes, or chemical conversion during storage. These attributes should be linked to critical process parameters such as raw-material grade, order of addition, mixing or homogenization energy, solvent removal, purification, drying, and sterilization. This control strategy follows the quality-by-design and risk-management principles described in ICH Q8(R2), ICH Q9(R1), and ICH Q10 and should be adapted to the U.S. Food and Drug Administration’s 2022 guidance for drug products containing nanomaterials.

### 6.3. CMC and Regulatory Progression

Regulatory planning should be anchored to a single clinically intended product rather than a broad platform label. Its quality target product profile should specify route, dosage form, release pattern, and the function of any targeting ligand or active carrier. Product classification and dossier scope would depend on whether a carrier, scaffold, siRNA, or co-delivered agent contributes an additional mode of action, for which early consultation with the relevant authority may be warranted. Before first-in-human evaluation, a candidate would generally require a defined manufacturing process, qualified raw materials, clinical-lot specifications and stability data, route-appropriate microbiological controls, and assays capable of quantifying total and free polydatin. These expectations are consistent with the product and process understanding, risk management, and pharmaceutical quality system principles described in ICH Q8(R2), ICH Q9(R1), and ICH Q10, together with the FDA’s 2022 guidance for drug products containing nanomaterials.

Manufacturing changes during scale-up should be evaluated through analytical comparability; changes in particle-size distribution, loading, release, or degradation may require additional pharmacokinetic, distribution, or toxicology bridging. Complex co-delivery systems also require separate control of siRNA, 4-PBA, gold, extracellular-vesicle, or ligand components and justification of each component’s contribution. The CMC package should mature with trial phase, progressing from qualified methods and provisional acceptance limits to validated assays, process validation, commercial-scale reproducibility, and shelf-life specifications [115]. For nanomaterial- or liposome-based candidates, the U.S. FDA guidance documents on drug products containing nanomaterials (2022) and liposome drug products (2018) provide additional product-specific considerations. These elements provide the product definition needed to interpret subsequent human evidence.

## 7. Clinical Translation

Against this product-development framework, human evidence for polydatin comprises oral combination products, topical preparations, and a registered intravenous study. A randomized multicenter study evaluated a palmitoylethanolamide/polydatin combination containing 20 mg polydatin twice daily for 12 weeks in patients with irritable bowel syndrome [116]. A placebo-controlled pilot study and a subsequent meta-analysis also evaluated micronized palmitoylethanolamide/trans-polydatin combinations in endometriosis-related pain, although these data do not isolate the contribution of polydatin [117,118]. Another study applied creams containing 0.8% or 1.5% polydatin twice daily for six months to manage cutaneous adverse reactions associated with epidermal growth factor receptor inhibitors [119]. A phase II study of intravenous polydatin for traumatic, hemorrhagic, or septic shock planned to enroll 240 participants and administer 200 mg over two hours once daily for five days. ClinicalTrials.gov currently lists the study status as unknown; the record was last verified in January 2013 as not yet recruiting, and no results have been posted [ClinicalTrials.gov NCT01780129; accessed 26 August 2026].

Taken together, these studies establish that polydatin-containing interventions have entered human evaluation through oral, topical, and intravenous routes. Although none assessed a polydatin nanocarrier, comparative pharmacokinetic data are available for oral formulations that increase systemic polydatin exposure [67,71], while local matrices provide sustained release and targeted or responsive platforms are supported mainly by cellular uptake and fluorescence-based distribution [23,25,75]. Further development should prioritize clinically feasible routes, controls receiving free polydatin by the same route and at a matched dose, quantitative measurement of polydatin and relevant metabolites at intended sites of action, and experimental designs linking exposure to mechanism-specific pharmacodynamic and safety endpoints. These requirements align with translational nanomedicine frameworks emphasizing reproducible product characterization, relevant preclinical models, and scalable manufacturing [120,121].

The reported rodent bioavailability gains should not be interpreted as estimates of human exposure. The three direct oral comparisons used different rat doses (10, 50, and 100 mg/kg), formulations, sampling designs, and analytical reporting, whereas the only human bioanalytical study evaluated conventional tablets and yielded sparse measurements of parent polydatin. Cross-study ratios therefore cannot bridge species or products. A human program should instead compare the intended clinical formulation with a route- and dose-matched free-polydatin reference and quantify parent polydatin, resveratrol, and major glucuronide and sulfate conjugates over a prespecified sampling window.

Body-surface-area conversion can provide a provisional human-equivalent dose for planning, but it is not a clinical dose recommendation and does not account for formulation-dependent absorption, metabolite formation, or carrier disposition [122]. This limitation should be considered alongside the body-surface-area approach described in the U.S. FDA’s 2005 guidance on estimating the maximum recommended starting dose in initial adult volunteer studies. Starting-dose selection should integrate route-appropriate repeat-dose toxicology, local tolerance, systemic exposure margins, and the pharmacological contribution of every active carrier or co-delivered component. For oral candidates, exposure matching is more informative than nominal mg/kg conversion; for injectable or local products, carrier burden, infusion or application volume, release rate, and tissue exposure require separate justification.

Early PK/PD studies should prespecify analytes and mechanism-linked biomarkers. Plasma polydatin, resveratrol, and principal conjugates should be measured alongside formulation-relevant free and total drug where feasible. Biomarkers should match the indication: lactate clearance, vasopressor requirement, and Sequential Organ Failure Assessment trajectories for shock; serum creatinine, cystatin C, and urinary injury markers for renal disease; pulmonary function, imaging, and fibrosis markers for lung disease; and endothelial function, perfusion, or neurovascular imaging for vascular indications. These endpoints should be analyzed against measured exposure rather than assigned dose alone. This exposure-response approach is consistent with the dose–response principles described in ICH E4.

We propose a staged roadmap beginning with a single development candidate supported by batch-reproducible CMC, stability, route-appropriate repeat-dose toxicology, and comparative preclinical PK/PD. Phase I or an early patient study, as appropriate to the indication, would evaluate single and multiple doses, tolerability, complete analyte pharmacokinetics, and formulation-specific risks; food-effect testing would be relevant for oral products and infusion reactions for parenteral products. A Phase Ib/IIa proof-of-mechanism study could then test an indication-specific biomarker and exposure-response hypothesis before larger Phase II efficacy trials. This progression is consistent with the nonclinical-to-clinical expectations described in ICH M3(R2) and the quality-by-design principles for clinical studies in ICH E8(R1). The unupdated status and absence of posted results for NCT01780129 prevent that registry record from serving as a human exposure or efficacy benchmark.

Under this sequence, a nanocarrier should advance only if it offers a measurable advantage over a simpler non-nano formulation at a clinically feasible dose and route. A targeted system would additionally require quantitative drug distribution or target-cell exposure rather than fluorescence alone. These criteria also frame the broader research priorities below.

## 8. Future Perspectives and Research Gaps

**Figure 5:**
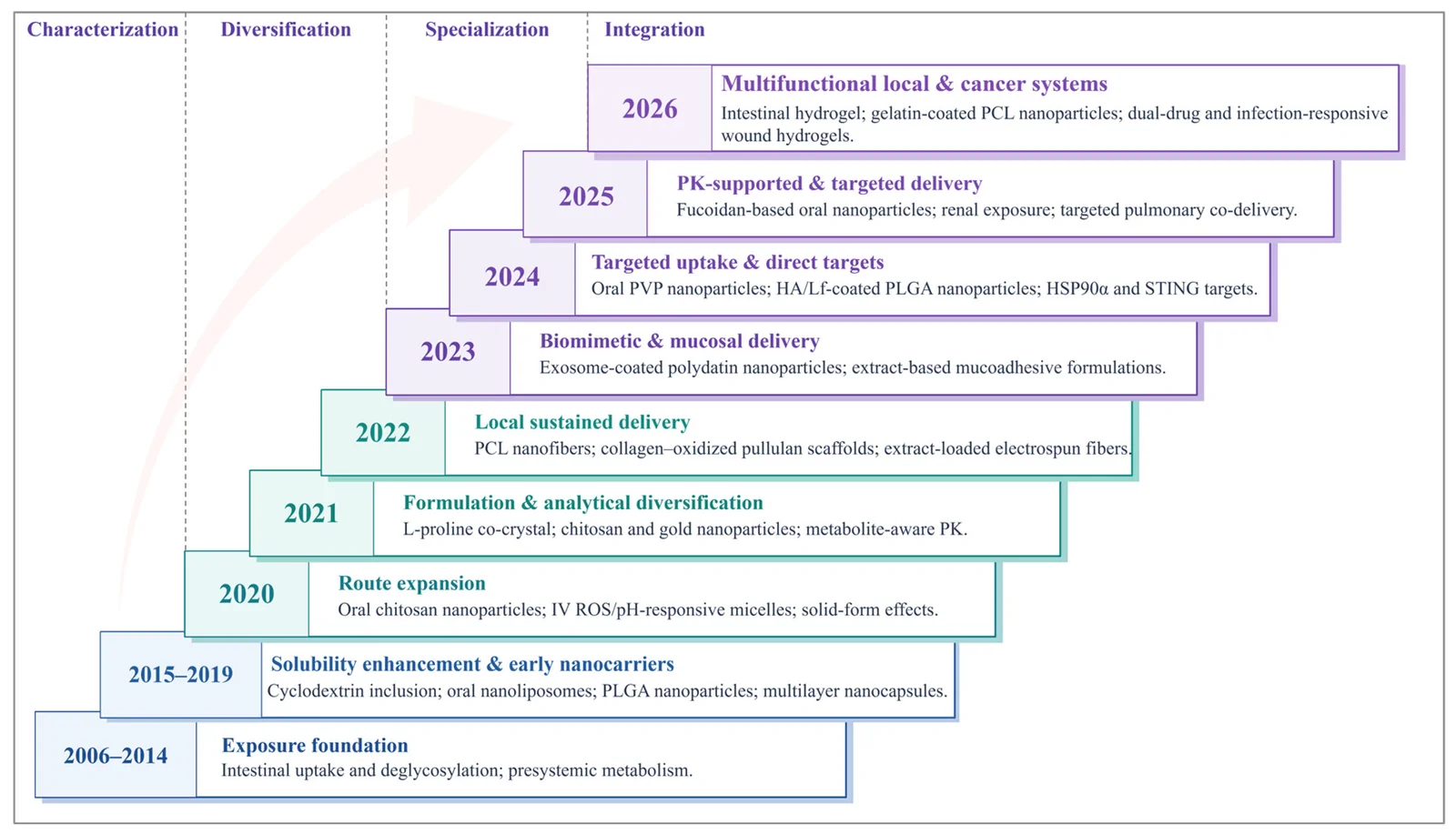
Key milestones in polydatin pharmacology, formulation, and delivery research from 2006 to 2026. The timeline summarizes four broad phases of development. The characterization phase established intestinal uptake, deglycosylation, presystemic metabolism, and limited oral exposure. Diversification involved solubility-enhancing strategies, early nanocarriers, expansion of administration routes and carrier materials, and metabolite-aware pharmacokinetic analysis. Specialization introduced sustained local delivery, biomimetic and mucosal formulations, receptor-associated cellular uptake, and direct-target pharmacology. Integration is represented by systems combining pharmacokinetic or tissue-exposure assessment with targeted co-delivery, responsive release, or multifunctional activity in intestinal, pulmonary, wound, and cancer models. The timeline is milestone-based and not drawn to scale. Non-nanoscale solid-state strategies and botanical-extract formulations are included as complementary formulation approaches. HA, hyaluronic acid; HSP90α, heat shock protein 90 alpha; IV, intravenous; Lf, lactoferrin; PCL, polycaprolactone; PK, pharmacokinetics; PLGA, poly(lactic-co-glycolic acid); PVP, polyvinylpyrrolidone; ROS, reactive oxygen species; STING, stimulator of interferon genes.

Polydatin is a chemically defined stilbenoid glucoside whose reported preclinical actions involve redox regulation, mitochondrial and endoplasmic reticulum homeostasis, inflammatory signaling, regulated cell death, autophagy, and mitophagy. Its glucose moiety distinguishes polydatin from resveratrol in physicochemical properties, intestinal handling, and metabolic fate. However, physical-form-dependent dissolution, intestinal hydrolysis, phase-II metabolism, and low oral bioavailability complicate the achievement of predictable exposure [14,15,36]. Delivery systems can therefore serve functions beyond solubility enhancement by modifying release, systemic exposure, tissue distribution, cellular uptake, and local residence. Because many reported mechanisms involve intracellular signaling proteins and organelles, total tissue accumulation alone does not establish pharmacologically relevant delivery (Figure 5).

The available evidence demonstrates several formulation-specific advantages, although their strength varies across administration routes and disease models. Oral nanoliposomes and fucoidan-based nanoparticles increased systemic polydatin exposure, and the fucoidan systems linked pharmacokinetic data and limited tissue-distribution measurements with functional, histological, and molecular responses in cisplatin-induced kidney injury [67,71,72]. Dose-matched studies also reported stronger pharmacodynamic responses than free polydatin in diabetes, intestinal injury, and developing pulmonary fibrosis [21,69,70]. Local matrices sustained polydatin release and supported tissue-repair responses, whereas targeted and responsive systems modified cellular uptake or release under defined experimental conditions [23,25,75]. For intravenous, local, targeted, and responsive systems, however, quantitative measurement of polydatin in target tissues and relevant cell populations remains uncommon. Pharmacological responses to active carriers and multicomponent formulations must also be interpreted as effects of the complete formulation unless appropriate component controls are included. The current evidence therefore distinguishes successful formulation construction from demonstrated changes in polydatin exposure and from evidence that those changes strengthen mechanism-related pharmacodynamic responses.

Rational carrier and combination design should begin with a defined route-specific barrier rather than with material novelty alone. Carrier composition, release trigger, ligand, and co-delivered agent should each have a prespecified function that can be tested with free-polydatin, blank-carrier, ligand-free, and component-specific controls. Multicomponent systems should be advanced only when the combined design produces an interpretable exposure or pharmacodynamic advantage that cannot be achieved by a simpler formulation. This approach would reduce redundant carrier variants and make formulation selection more closely aligned with the intended clinical indication.

Further development should prioritize formulations supported by reproducible evidence of route-appropriate release, altered exposure, or advantages over dose- and route-matched free polydatin. Simultaneous measurement of polydatin, resveratrol, and major conjugated metabolites in plasma and target tissues would clarify how delivery systems alter disposition and metabolic conversion. Studies proposing intracellular delivery should additionally measure cellular polydatin exposure or target engagement and relate these findings to mechanism-specific pharmacodynamic endpoints. Repeated-dose safety, immune responses, material degradation and clearance, batch reproducibility, storage stability, and scalable manufacturing should be evaluated for the formulations selected for further development. No study identified in this review has evaluated a polydatin delivery system in a cardiovascular disease model, despite preclinical evidence for free polydatin in endothelial dysfunction, cardiac remodeling, and atherosclerosis [38,60,96,110]. Delivery studies in neurovascular disorders are likewise sparse despite a broader preclinical literature on free polydatin [6]. Extending cardiovascular and neurovascular delivery research should address a defined exposure, cellular-delivery, blood-brain-barrier, or targeting limitation rather than rely on pharmacological activity alone. Machine-learning approaches may eventually assist formulation optimization and prediction of critical quality attributes, but reliable application will require standardized formulation, biodistribution, PK/PD, and safety datasets that are not yet available for polydatin delivery systems [123,124,125]. Human studies have evaluated oral combinations and topical preparations containing polydatin [116,119], while the intravenous shock trial remains of unknown status and without posted results [ClinicalTrials.gov NCT01780129; accessed 26 August 2026]; no polydatin nanocarrier has yet undergone clinical evaluation. Candidate selection should therefore favor formulations that combine reproducible manufacturing, route-specific exposure, mechanism-related pharmacodynamic effects, and an acceptable safety profile, with a measurable advantage over free polydatin.

## 9. Conclusions

Polydatin delivery research has progressed from solubility-oriented formulation to systems intended to modify exposure, release, distribution, cellular uptake, and local residence. The strongest comparative evidence remains a small oral pharmacokinetic subset; most injectable, targeted, responsive, and local platforms are supported primarily by formulation, imaging, or pharmacodynamic data rather than quantitative drug exposure. Translation will therefore depend on selecting a route-appropriate product, demonstrating reproducible manufacture and safety, measuring polydatin and its metabolites, and showing an exposure-linked advantage over matched free drug. These priorities provide a practical basis for advancing the most credible candidates toward clinical evaluation.

## Figures and Tables

**Table 1:** Pharmacological mechanisms of polydatin and their implications for delivery design.

Mechanistic Category	Strongest Evidence or Control	Representative Molecular and Functional Findings	Relevance to Delivery Design
Redox regulation and mitochondrial function	Membrane-biophysical assays; graded dose–response studies; mitochondrial permeability-transition testing; SIRT3 and Nrf2 pathway manipulation	Lower lipid peroxidation and NOX2/NOX4 signaling; preserved mitochondrial potential and ATP; increased antioxidant-response signaling	Requires adequate tissue and intracellular exposure; organ or cell uptake should be measured rather than inferred from downstream antioxidant effects.
Inflammatory and innate immune signaling	Lyn/Syk kinase assays; SIRT1/p38 inhibition; primary human neutrophils; STING binding, knockdown, and overexpression	Reduced mast-cell, microglial, NET, NF-κB, and STING/TBK1 responses, with lower cytokine and extracellular-matrix production	Supports cell-selective delivery, but pathway-marker changes alone do not establish carrier targeting or target engagement.
Inflammasome signaling and regulated cell death	HSP90α binding and knockout; inflammasome controls; ferrostatin-1 and Nrf2 knockdown	Reduced NLRP3-complex stability and pyroptotic signaling; context-dependent suppression of ferroptosis through Nrf2/SLC7A11/GPX4	Formulation studies should link quantified exposure to pathway-specific endpoints and distinguish carrier effects from polydatin effects.
Autophagy, mitophagy, and endoplasmic reticulum stress	Autophagy inhibition; SIRT6 or SIRT1 silencing; Parkin knockout; mitochondrial-fission controls	Context-dependent increases in autophagy or mitophagy with reduced endoplasmic reticulum stress, apoptosis, and organ injury	Intracellular release and organelle access may be critical; tissue accumulation alone does not demonstrate delivery to the relevant subcellular compartment.

Note: This table summarizes the mechanistic categories most relevant to delivery design. Complete study-level models, exposures, mechanistic controls, molecular changes, functional outcomes, and study identifiers are provided in Supplementary Table S5. Abbreviations: ATP, adenosine triphosphate; GPX4, glutathione peroxidase 4; HSP90α, heat shock protein 90 alpha; NET, neutrophil extracellular trap; NF-κB, nuclear factor kappa B; NLRP3, NLR family pyrin domain containing 3; NOX, NADPH oxidase; SIRT, sirtuin; SLC7A11, solute carrier family 7 member 11; STING, stimulator of interferon genes.

**Table 2:** Direct within-study pharmacokinetic comparisons of free and formulated polydatin.

Formulation	Matched Design	Cmax: Free vs. Formulated	AUC: Free vs. Formulated	Other PK/Distribution	Supported Inference	Key Limitation	Reference
PLD-loaded liposome	Rats; oral 10 mg/kg; HPMC suspension vs. liposome; *n* = 6/group	1562.7 +/− 453.2 vs. 1898.3 +/− 376.5 ng/mL; not marked significant	AUC0-inf 5387.4 +/− 527.4 vs. 15,241.5 +/− 945.5 ng h/mL (2.83-fold)	t1/2 2.78 +/− 0.89 vs. 18.36 +/− 3.54 h; MRT 6.8 +/− 2.3 vs. 32.2 +/− 3.4 h	Increased systemic exposure and prolonged apparent disposition	Figure caption states intravenous administration, whereas the Methods and main text specify oral administration; no tissue-targeting data	[67]
Fu/Cs Po nanoparticles	Rats; oral 50 mg/kg; free Po vs. nanoparticles; *n* = 3/group	1114.4 +/− 142.1 vs. 4202.7 +/− 407.2 ng/mL (3.77-fold)	AUC0-24 10,781.4 vs. 25,091.1 in the authors’ reported unit (2.33-fold)	Tmax 2.5 vs. 2 h; MRT 8.5 vs. 6.7 h; tissue Po measured at 2 h	Increased systemic exposure and higher Po in several organs at one time point	Questionable reported AUC unit; Multi-organ enrichment does not prove active kidney targeting	[71]
Fu-4-PBA/Po nanoparticles	Rats; oral 100 mg/kg; free Po vs. nanoparticles; *n* = 3/group	18,347.262 vs. 39,928.965 ng/mL (2.18-fold)	AUC0-24 198,270.4167 vs. 398,166.85 microg/L h (2.01-fold)	Tmax 1.5 vs. 2 h; MRT 15.7 vs. 23.8 h; tissue Po measured in rats	Increased systemic exposure; carrier-level P-selectin/ER targeting supported in vitro	Active 4-PBA; mouse DiR imaging and rat drug assay were discordant; no confirmed Po kidney targeting	[72]

Values are reported as free polydatin versus formulated polydatin. AUC units are reproduced from the source articles because two reports contain internal unit inconsistencies. An AUC increase was interpreted as increased systemic exposure; target-organ delivery required direct tissue-polydatin measurement and appropriate controls. AUC, area under the plasma concentration-time curve; Cmax, maximum plasma concentration; MRT, mean residence time; PK, pharmacokinetics; t1/2, elimination half-life.

**Table 3:** Oral delivery and formulation-enabling strategies for polydatin.

Delivery System (Composition)	Model and Comparators	Route/Regimen	Principal Finding and Evidence Boundary	Reference
Polydatin/Malt-β-CD inclusion complex Composition: Polydatin; 6-O-α-maltosyl-β-cyclodextrin	Cell-free formulation study Comparators: Polydatin; Malt-β-CD; physical mixture; inclusion complex	1:1 host-guest complex; phase-solubility and solid-state analyses	Mechanistic/functional: Polydatin crystallinity ↓; docking predicted ring-B insertion toward the narrow rim and seven stabilizing hydrogen bonds Outcomes: Aqueous solubility ↑; 1:1 inclusion complex formed	[65]
Trans-polydatin-CD inclusion complexes (trans-polydatin-β-CD, -Me-β-CD, and -HP-β-CD) Composition: Polydatin with β-CD, methyl-β-CD, or HPβCD	Cell-free formulation study Comparators: Free trans-polydatin; trans-polydatin-β-CD; trans-polydatin-Me-β-CD; trans-polydatin-HP-β-CD	Cyclodextrins tested at 10 mM; solution and solid-state studies	Mechanistic/functional: Antioxidant activity ↑ after complexation Outcomes: Apparent solubility ↑ approximately 6.4-, 7.9-, and 9.0-fold; thermal and photochemical stability ↑	[29]
Polydatin-L-proline co-crystal (PD-L-Pro) Composition: Polydatin and L-proline (1:3)	Dissolution study; A549 and HEK-293 cells Comparators: Polydatin versus PD-L-Pro; resveratrol formulations also tested	Dissolution at pH 7.0 and 37 °C; cell exposure for 24 h	Mechanistic/functional: MTT: A549 viability ↓; HEK-293 viability largely retained after 24 h Outcomes: Equilibrium solubility ↑ 15.8%; cytotoxicity retained in A549 cells with low HEK-293 toxicity	[66]
Polydatin solid forms (form A, form B, and amorphous form C) Composition: Anhydrous and trihydrate polydatin	Rat pharmacokinetics Comparators: Polydatin form A (anhydrate) versus form B (trihydrate)	Single oral dose, 150 mg/kg	Mechanistic/functional: Hydrogen-bond networks and crystal packing differed between forms A and B; amorphous form C converted to form A at room temperature Outcomes: Trihydrate: faster absorption and approximately 1.5-fold greater systemic exposure	[28]
Polydatin-loaded liposomes (PLD-loaded liposomes) Composition: Soybean lecithin, cholesterol, sodium cholate, isopropyl myristate, and polydatin	Rat pharmacokinetics Comparators: PLD-HPMC suspension versus PLD-loaded liposomes	Single oral dose, 10 mg/kg	Mechanistic/functional: Absorption mechanism not resolved. Outcomes: t_1/2_ approximately 2.8 to 18.4 h; AUC approximately 3-fold ↑; relative bioavailability 282.9%	[67]
Ultrasound-assisted liposome-encapsulated piceid Composition: L-α-phosphatidylcholine/cholesterol (2:1) and piceid	Cell-free process optimization Comparators: Box–Behnken combinations of lipid content, ultrasound power, and treatment time	5 mg piceid; lipid 120–180 mg; ultrasound 90–150 W; 18–30 min	Mechanistic/functional: Lipid content and ultrasound time significantly affected EE; lipid content affected loading; ultrasound power was not significant in the fitted models Outcomes: Optimized EE 75.82% and loading 2.37%; no biological or PK evaluation	[68]
Polydatin-loaded chitosan nanoparticles (PD-CSNPs) Composition: Chitosan, tripolyphosphate, and polydatin	Nicotinamide/STZ-induced type 2 diabetes in rats Comparators: Normal; diabetic; free polydatin; blank CSNPs; PD-CSNPs; metformin	Oral gavage, polydatin equivalent 50 mg/kg once daily for 28 days	Mechanistic/functional: Glycemic indices, oxidative stress, and inflammatory markers ↓; antioxidant enzymes ↑ Outcomes: Fasting glucose, HbA1c, insulin resistance, and serum lipid profile improved more than with free polydatin	[21]
Polydatin-loaded chitosan nanoparticles (POL-NPs) Composition: Chitosan, tripolyphosphate, and polydatin	Early diabetic nephropathy in rats Comparators: Normal; diabetic; free polydatin (POL); POL-NPs	Oral gavage, polydatin equivalent 50 mg/kg once daily for 4 weeks	Mechanistic/functional: AGEs, LPO, NF-κB, COX-2, TNF-α, IL-6, IL-18 ↓; IL-10 and antioxidant enzymes ↑ Outcomes: Glycemic and renal function indices improved more than with free polydatin	[85]
Polydatin-loaded chitosan nanoparticles (PD-CSNPs) Composition: Chitosan, tripolyphosphate, and polydatin	Nicotinamide/STZ-induced diabetic liver injury in rats Comparators: Normal; diabetic; free polydatin; blank CSNPs; PD-CSNPs; metformin	Oral gavage, polydatin equivalent 50 mg/kg once daily for 4 weeks	Mechanistic/functional: GLUT2 and glucokinase ↑; TNF-α, IL-1β, LPO, transaminases ↓; antioxidant enzymes ↑ Outcomes: Hepatic glucose metabolism, antioxidant status, and histology improved more than with free polydatin	[84]
Polyvinylpyrrolidone-polydatin nanoparticles (PVP-PD) Composition: Polyvinylpyrrolidone and polydatin	Oxaliplatin-induced intestinal injury in mice; intestinal epithelial cells Comparators: Control; oxaliplatin; PVP; free polydatin; PVP-PD; N-acetylcysteine	Oral 50 or 100 mg/kg once daily for 7 days; oxaliplatin on days 3–6	Mechanistic/functional: ROS, γ-H2AX, cGAS, STING, IL-1β, TNF-α ↓; mitochondrial indices ↑ Outcomes: Body weight, colon length, crypt/villus morphology, goblet cells, and mucosal integrity preserved	[70]
COS-coated polydatin-loaded PLGA nanoparticles (COS-coated PD/PLGA NPs) Composition: PLGA core, chitooligosaccharide coating, and polydatin	Bleomycin-induced pulmonary injury/fibrosis in rats Comparators: Control; bleomycin; free polydatin; blank COS-coated PLGA NPs; COS-coated PD/PLGA NPs	Oral polydatin equivalent 25 mg/kg once daily; dosing began before bleomycin and continued through day 17	Mechanistic/functional: α-SMA, TGF-β, LPO, TNF-α, IL-1β ↓; antioxidant defenses ↑ Outcomes: Lung architecture and collagen deposition improved more than with free polydatin	[69]
Fucoidan/carboxymethyl chitosan polydatin nanoparticles (Fu/Cs Po NPs) Composition: Fucoidan, carboxymethyl chitosan, and polydatin	Rat PK; cisplatin-induced acute kidney injury Comparators: PK: free Po versus Fu/Cs Po NPs; efficacy: control, cisplatin, free Po, Fu/Cs NPs, Fu/Cs Po NPs	PK: single oral 50 mg/kg; efficacy: oral 100 mg/kg beginning 2 h before cisplatin and continued daily	Mechanistic/functional: ROS, γ-H2AX, cytosolic mtDNA, cGAS-STING signaling ↓ Outcomes: Serum creatinine, BUN, tubular injury, oxidative stress, and inflammation ↓	[71]
Fucoidan-4-PBA/polydatin nanoparticles (Fu-4-PBA/Po NPs) Composition: Fucoidan, 4-phenylbutyric acid, and polydatin	Rat PK; cisplatin-induced acute kidney injury Comparators: Free Po; Fu-4-PBA; Fu-4-PBA/Po NPs at 50/100 mg/kg; P-selectin inhibitor condition	PK: single oral 100 mg/kg; efficacy: 50 or 100 mg/kg beginning 2 h before cisplatin, once daily for 4 days	Mechanistic/functional: PERK/eIF2α/ATF4/CHOP, DNA damage, cGAS-STING signaling ↓ Outcomes: Plasma exposure and renal outcomes improved; direct tissue Po was highest in liver, and effects reflect both delivery and active 4-PBA	[72]
Polydatin-encapsulated PLGA nanoparticles (POL-PLGA-NPs) Composition: PLGA and polydatin	DMBA-induced buccal carcinogenesis in hamsters Comparators: Control; DMBA; POL-PLGA-NPs at three doses; PLGA-NP-only condition	Oral 7.5, 15, or 30 mg/kg three times weekly during a 14-week DMBA protocol	Mechanistic/functional: LPO and proliferation-related markers ↓; antioxidant defenses ↑ Outcomes: Tumor incidence and burden ↓ in a dose-related manner	[24]
Polydatin graphene hydrogel (PD@GO-PAA) Composition: Graphene oxide-polyacrylic acid hydrogel and polydatin	Radiation-induced intestinal injury in FHs 74Int cells and mice Comparators: IR + vehicle; IR + GO-PAA; IR + free polydatin; IR + PD@GO-PAA; nonirradiated controls in tissue analyses	Single gavage 0.5 h before irradiation: polydatin 500 μg and/or dry hydrogel 2 mg; 18 Gy survival or 14 Gy tissue protocol	Mechanistic/functional: Epithelial viability and Ki67-positive crypt regeneration ↑ Outcomes: Survival, leukocyte count, villus length, crypt number, and intestinal histology improved versus free polydatin	[73]

Note: Author-defined formulation abbreviations are retained; full evidence classifications and targeting appraisals are provided in Supplementary Table S4. Upward (↑) and downward (↓) arrows indicate increases and decreases, respectively.

**Table 4:** Injectable, targeted, and exploratory polydatin delivery systems.

Delivery System (Composition)	Model and Comparators	Route/Regimen	Principal Finding and Evidence Boundary	Reference
Polydatin amphiphilic chitosan nanocarrier (NPD) Composition: Chitosan, stearic acid, Tween 80, tripolyphosphate, and polydatin	AlCl_3_-induced Alzheimer-like model in rats Comparators: Sham; AlCl_3_; donepezil; blank ACN; NPD 10 mg/kg; NPD 20 mg/kg	Intraperitoneal once daily for 14 days; AlCl_3_ 100 mg/kg; donepezil 10 mg/kg; blank ACN 2 mg/kg	Mechanistic/functional: MMP-9 and nitrite ↓; MMP-2, CAT, GSH ↑ Outcomes: Memory/behavioral measures and hippocampal neuronal survival improved dose dependently	[86]
ROS- and pH-responsive polydatin-loaded micelle (PD-MC) Composition: PEG-P(PBEM-co-DPA) block copolymer and polydatin	CCl_4_-induced liver fibrosis in mice; hepatocytes, macrophages, and hepatic stellate cells Comparators: Model; IV blank micelle (MC); IV PD-MC; IP free polydatin; healthy controls	Tail vein every other day for 3 weeks: carrier 40 mg/kg containing polydatin 2.5 mg/kg; free polydatin IP 2.5 mg/kg	Mechanistic/functional: ROS, TLR4/NF-κB, NOX4, inflammatory macrophage activity, α-SMA, collagen synthesis ↓ Outcomes: Hepatocyte apoptosis, stellate cell activation, collagen deposition, and fibrosis ↓	[23]
Exosome-coated polydatin nanoparticles (exo-PD) Composition: Human amniotic fluid stem-cell exosomes and polydatin nanoparticles	Radiation-induced intestinal injury in cells and mice Comparators: Control; irradiation; free polydatin; exosomes; exo-PD	Intraperitoneal immediately after irradiation; polydatin 20 mg/kg and exosomes 2 mg/kg	Mechanistic/functional: PI3K/AKT signaling and mitochondrial function ↑; IL-1α, IL-6, γ-H2AX, senescence ↓ Outcomes: Survival, leukocyte recovery, diarrhea, villus/crypt injury, and mitochondrial indices improved	[22]
Polydatin-gold nanoparticle formulations (PD-AuNPL, PD-AuNPH, and PD-Dox-AuNP) Composition: Gold nanoparticles, polydatin, and optional doxorubicin	Ehrlich ascites carcinoma-bearing mice Comparators: Tumor control; free PD; Dox; AuNP; PD-AuNPL; PD-AuNPH; Dox-AuNP; PD-Dox-AuNP	Intraperitoneal for 21 days; polydatin 20 mg/kg, AuNP 5 or 10 ppm, doxorubicin 2 mg/kg	Mechanistic/functional: Oxidative, inflammatory, apoptotic, and tumor-associated biochemical markers modulated Outcomes: Tumor-related and biochemical endpoints improved, especially with doxorubicin-containing combinations	[74]
SPFPT-modified siImp7/polydatin LNP (SPFPT@siImp7/PD-LNP) Composition: Lipid nanoparticle, SPFPT peptide, polydatin, and siRNA targeting importin 7	Ventilator-induced lung injury in mice; alveolar epithelial cells Comparators: siImp7/PD-LNP; GTCRV@siImp7/PD-LNP; SPFPT@PD-LNP; SPFPT@siImp7-LNP; SPFPT@siImp7/PD-LNP; budesonide	Intratracheal after 4 h mechanical ventilation; targeting study used 5 μg Cy5-siRNA and 2-h assessment	Mechanistic/functional: p38 nuclear translocation, NF-κB, TNF-α, IL-6, HMGB1 ↓ Outcomes: Pulmonary edema, neutrophil infiltration, BALF inflammatory markers, and histological injury ↓	[75]
HA110/Lf-coated polydatin/PLGA nanoparticles (F9) Composition: PLGA, polydatin, hyaluronic acid, and lactoferrin	A549 lung cancer cells Comparators: Free PD; PD/PLGA NPs (F1); Lf-coated NPs (F4); HA32/Lf-coated NPs (F7); HA110/Lf-coated NPs (F9); blank carriers; CD44 blockade	In vitro exposure up to 72 h	Mechanistic/functional: ROS generation and cytotoxicity ↑ in A549 cells Outcomes: Supports receptor-associated internalization in vitro; no in vivo tumor targeting	[76]
Six-layer polydatin-loaded nanocapsules (NCPD6) Composition: AOT nanoemulsion core with poly-L-lysine/poly-L-glutamic acid multilayer shell	LPS-exposed rat hippocampal organotypic cultures Comparators: Vehicle; LPS; free PD; empty NC6; NCPD6 with/without LPS	Free polydatin 5–50 μM or loaded capsules 5 × 10^10^–2 × 10^11^ particles/mL; 1-h pretreatment, then LPS for 24/48 h	Mechanistic/functional: Cell death, TNF-α, IL-1β, NO, iNOS ↓; inflammatory signaling attenuated Outcomes: Nanocapsulation preserved the anti-inflammatory and cytoprotective effects of free polydatin	[87]
Polydatin-loaded chitosan nanoparticles (PD-CS-NPs) Composition: Chitosan-tripolyphosphate nanocapsules and polydatin (1:1 *w*/*w*)	HER2-overexpressing SKBR3 breast cancer cells Comparators: Untreated/DMSO; chitosan; empty CS-NPs; free PD; PD-CS-NPs	200 or 400 μg/mL for 24, 48, or 72 h	Mechanistic/functional: Actin depolymerization and chromatin condensation at 72 h Outcomes: Proliferative capacity ↓ approximately 40% and 60% at 200 and 400 μg/mL after 72 h	[88]
Polydatin-loaded gelatin-coated PCL nanoparticles (PPG-NPs) Composition: PCL, gelatin type A, Pluronic F127, and polydatin	A549, radioresistant A549, and normal L132 cells; 2D cultures and 3D spheroids Comparators: Untreated; blank PG-B NPs; free polydatin; PPG-NPs	50–200 μg/mL; most mechanistic assays at 200 μg/mL for 72 h; spheroids treated for 72 h	Mechanistic/functional: ROS, DNA damage, γ-H2AX, p53, apoptosis, E-cadherin ↑; Ki67, migration, EGFR, AKT1, mTOR ↓ Outcomes: Greater reduction in viability, clonogenicity, migration, and radioresistant spheroid growth than free polydatin	[89]
Chitosan-coated polydatin@ZIF-8 (PD@ZIF-8/CS) Composition: ZIF-8, polydatin, and chitosan	Cell-free formulation and antibacterial assays Comparators: ZIF-8, PD@ZIF-8, and PD@ZIF-8/CS; free-component controls as reported	In vitro release over 72 h; antibacterial testing against *S. aureus* and *E. coli*; no animal dosing	Formulation: 91.34% drug-loading efficiency; 92.5% release at pH 5.0 over 72 h; chitosan coating reduced burst release Boundary: Antibacterial activity was demonstrated in vitro; no in vivo exposure, efficacy, or selective targeting evidence	[90]

Note: Upward (↑) and downward (↓) arrows indicate increases and decreases, respectively.

**Table 5:** Local delivery systems containing defined polydatin.

Delivery System (Composition)	Model and Comparators	Route/Regimen	Principal Finding and Evidence Boundary	Reference
Polydatin-incorporated PCL nanofibers (PD-PCL) Composition: Electrospun polycaprolactone and polydatin	Mesenchymal stem-cell osteogenic differentiation Comparators: PCL; PD-PCL; PCL plus free polydatin	In vitro; free-polydatin condition 48 μM; release followed for 14 days	Mechanistic/functional: Osteogenic differentiation and matrix-related markers ↑ Outcomes: Supported osteogenic differentiation; no implantation or local exposure study	[77]
Polydatin-loaded collagen/oxidized pullulan scaffold (Col-OxP3-Po) Composition: Collagen, oxidized pullulan, and polydatin	Normal and diabetic excisional wounds in rats Comparators: Untreated; collagen; Col-OxP3; Col-OxP3-Po	Scaffold prepared with 30 mg polydatin; follow-up to day 16 in normal and day 21 in diabetic rats	Mechanistic/functional: Collagen deposition and tissue-repair markers ↑ Outcomes: Wound closure accelerated; complete closure by day 21 in diabetic wounds	[25]
OHA/CS-pC/TMP/PD hydrogel Composition: Oxidized hyaluronic acid, catechol-chitosan, tetramethylpyrazine, and polydatin	Diabetic excisional wounds Comparators: Untreated; OHA/CS-pC hydrogel; OHA/CS-pC/TMP/PD hydrogel	Polydatin 0.4 mg/mL and tetramethylpyrazine 0.068 mg/mL; 14-day follow-up	Mechanistic/functional: Markers of angiogenesis and matrix remodeling modulated Outcomes: Nearly complete wound closure by day 14; individual drug contributions unresolved	[78]
DAP1 hydrogel containing MZCP Composition: Polydatin-loaded Cu-doped ZIF-8 nanozyme in aminated dextran/dialdehyde glucan hydrogel	Infected diabetic oral ulcers in rats Comparators: PBS; nanoparticle-free DAP0 hydrogel; complete DAP1 hydrogel	Local application; follow-up to day 6	Mechanistic/functional: Early antibacterial ROS generation proposed; later antioxidant and repair responses observed Outcomes: Bacterial burden ↓; ulcer closure, angiogenesis, and tissue repair ↑	[81]
Carrier-free mangiferin-polydatin co-assembled hydrogel Composition: Mangiferin and polydatin assembled through π-π stacking and hydrogen bonding	MRSA-infected diabetic wounds in rats Comparators: Not fully specified in the abstract; component-specific attribution not established	Topical wound dressing with continuous release; dose and follow-up schedule not stated in the abstract	Mechanistic/functional: Antibacterial and biofilm effects, ROS scavenging, M2-associated macrophage shift, and revascularization Outcomes: Wound repair accelerated; effects attributed to the complete binary hydrogel	[91]

Note: Author-defined formulation abbreviations are retained; full evidence classifications and targeting appraisals are provided in Supplementary Table S4. Upward (↑) and downward (↓) arrows indicate increases and decreases, respectively.

**Table 6:** Local botanical-extract formulations containing polydatin/piceid.

Delivery System (Composition)	Model and Comparators	Route/Regimen	Principal Finding and Evidence Boundary	Reference
PVP/HPβCD electrospun nanofibers (nanofiber no. 5) Composition: *Polygoni cuspidati* extract, PVP, and cyclodextrin	Cell-free dissolution/permeability study Comparators: Extract and isolated stilbenes versus extract-loaded nanofibers	Electrospun formulation; no animal dosing	Mechanistic/functional: Extract antioxidant and anti-inflammatory activity retained Outcomes: Polydatin and resveratrol solubility approximately 6-fold ↑; penetration coefficients ↑	[92]
PVP/HPβCD nanofiber tablets (TN) Composition: *Polygoni cuspidati* extract, PVP, and HPβCD	Cell-free local oral-mucosal formulation study Comparators: Tablets from nanofibers (TN); tablets from powder (TP); extract/formulation comparators	Local buccal/periodontal concept; no in vivo dosing	Mechanistic/functional: Extract antioxidant, anti-inflammatory, and antibacterial activity Outcomes: Complete but prolonged polydatin/resveratrol release and prolonged mucosal residence	[79]
HME extrudate-based mucoadhesive tablets (F1 and F3) Composition: *Polygoni cuspidati* extract with Kollidon VA64, Soluplus, or Kollicoat IR; HPMC in tablets	Cell-free dissolution, permeability, and mucoadhesion study Comparators: Extrudate-based tablets (F1 and F3); powder controls (F2 and F4)	Buccal formulation concept; no biological dosing	Mechanistic/functional: Amorphization, improved wetting, and molecular dispersion in Kollidon VA64 increased dissolution; HPMC supported mucoadhesion Outcomes: Kollidon VA64 formulation provided the best tabletability, release, and mucoadhesion	[93]
*Reynoutria japonica* extract-containing films (OW1-OW3; optimized OW2) Composition: Extract with HPMC/MC/NaCMC, pullulan, PVA, PVP, and glycerol	Cell-free film optimization Comparators: More than 80 formulations; placebo films; extract-containing films OW1-OW3; OW2 selected for release modeling	Solvent-cast local periodontal films; no biological dosing	Mechanistic/functional: Polymer viscosity and physical cross-linking regulated disintegration and mucoadhesion; piceid release was best fitted by the multidimensional model Outcomes: Sustained piceid and resveratrol release; formulation behavior depends on polymer composition	[80]

Note: Author-defined formulation abbreviations are retained; full evidence classifications and targeting appraisals are provided in Supplementary Table S4. Upward (↑) and downward (↓) arrows indicate increases and decreases, respectively.

**Table 7:** Pharmacological effects of polydatin delivery systems in disease models.

Disease Model	Delivery System	Principal Outcome and Evidence Boundary	Reference
Nicotinamide/streptozotocin-induced type 2 diabetes in rats	Polydatin-loaded chitosan nanoparticles (PD-CSNPs)	Improved glycemic control and insulin sensitivity; responses were generally greater than with free polydatin	[21]
Early diabetic nephropathy induced by high-fat diet and nicotinamide/streptozotocin in rats	Polydatin-loaded chitosan nanoparticles (POL-NPs)	FBG, HbA1c, BUN, serum creatinine and renal injury ↓; albumin ↑	[85]
Diabetic liver injury induced by nicotinamide/streptozotocin in rats	Polydatin-loaded chitosan nanoparticles (PD-CSNPs)	Hepatic glucose metabolism and glycogen storage ↑; ALT, AST and hepatic injury ↓	[84]
Full-thickness excisional wounds in diabetic rats	Polydatin-loaded collagen/oxidized pullulan scaffold (Col-OxP3-Po)	Collagen deposition, angiogenesis, re-epithelialization and wound closure ↑; closure by day 21	[25]
Full-thickness excisional wounds in diabetic mice	Oxidized hyaluronic acid/catechol-modified chitosan/tetramethylpyrazine/polydatin hydrogel (OHA/CS-pC/TMP/PD)	Angiogenesis, matrix formation and wound closure ↑; effects reflect the complete formulation	[78]
*Staphylococcus aureus*-infected oral ulcers in diabetic rats	DAP1 hydrogel incorporating polydatin-loaded Cu-doped ZIF-8 nanozymes (MZCP/DAP1)	Bacterial survival and residual wound area ↓; vascularization and repair ↑; complete-formulation effect	[81]
Cisplatin-induced acute kidney injury in mice	Fucoidan/carboxymethyl chitosan polydatin nanoparticles (Fu/Cs Po NPs)	AUC and renal exposure ↑; BUN, serum creatinine, tubular injury and inflammation ↓	[71]
Cisplatin-induced acute kidney injury in mice	Fucoidan-4-phenylbutyric acid/polydatin nanoparticles (Fu-4-PBA/Po NPs)	AUC and kidney exposure ↑; renal function and histological injury improved; complete-formulation effect	[72]
Radiation-induced intestinal injury in mice and irradiated HIEC-6 cells	Exosome-coated polydatin nanoparticles (exo-PD)	Villus length, crypt regeneration and survival ↑; senescence-related signal ↓; exosomes were biologically active	[22]
Oxaliplatin-induced intestinal injury in mice and NCM460 cells	Polyvinylpyrrolidone-polydatin nanoparticles (PVP-PD NPs)	Villus injury and goblet-cell loss ↓; 100 mg/kg nanoparticles outperformed dose-matched free polydatin	[70]
Radiation-induced intestinal injury in mice	Polydatin-loaded graphene oxide-polyacrylic acid hydrogel (PD@GO-PAA)	Intestinal sustained release, crypt number, villus length and survival ↑; body-weight and leukocyte loss ↓	[73]
Ventilator-induced lung injury in mice	SPFPT-modified lipid nanoparticles co-delivering importin 7 siRNA and polydatin (SPFPT@siImp7/PD-LNP)	Pulmonary edema, neutrophil infiltration and histological injury ↓; co-delivery outperformed either component alone	[75]
Bleomycin-induced pulmonary fibrosis in rats	Chitooligosaccharide-coated polydatin-loaded PLGA nanoparticles (COS-coated PD/PLGA NPs; F6)	Lung inflammation, collagen deposition and fibrotic remodeling ↓; evaluated during fibrosis development	[69]
CCl_4_-induced liver fibrosis in mice	ROS- and pH-dual-sensitive polydatin-loaded micelle (PD-MC)	Macrophage accumulation, hepatocyte apoptosis, collagen and hydroxyproline ↓; blank micelle also scavenged ROS	[23]
DMBA-induced buccal carcinogenesis in hamsters	Polydatin-encapsulated PLGA nanoparticles (POL-PLGA-NPs)	Tumor incidence, volume and burden ↓; nanocarrier advantage could not be isolated	[24]
Ehrlich ascites carcinoma in mice	Polydatin-gold nanoparticles (PD-AuNPL/PD-AuNPH) and polydatin/doxorubicin-gold nanoparticles (PD-Dox-AuNP)	Tumor weight and volume ↓; combination effects reflect polydatin, doxorubicin and AuNPs	[74]
A549 lung cancer cells	High-molecular-weight hyaluronic acid/lactoferrin-coated polydatin/PLGA nanoparticles (HA110/Lf-coated PD/PLGA NPs; F9)	A549 viability and IC_50_ ↓; evidence supports CD44-associated internalization in vitro	[76]
HER2-overexpressing SKBR3 breast cancer cells	Polydatin-loaded chitosan nanoparticles (PD-CS-NPs)	Proliferative capacity and cell attachment ↓; empty nanoparticles did not reduce viability	[88]
A549 and radioresistant A549 cells in 2D cultures and 3D spheroids	Polydatin-loaded gelatin-coated polycaprolactone nanoparticles (PPG-NPs)	DNA damage and growth inhibition ↑; migration ↓; greater responses in radioresistant cells and spheroids	[89]
LPS-exposed rat hippocampal organotypic cultures	Six-layer polyelectrolyte-shell polydatin nanocapsules (NCPD6)	Neuroinflammatory and cytotoxic responses↓	[87]
AlCl_3_-induced Alzheimer-like model in rats	Amphiphilic chitosan nanocarrier containing polydatin (NPD)	Learning, memory and behavioral outcomes improved; brain polydatin exposure was not measured	[86]
Saos-2 cells and human mesenchymal stem cells	Polydatin-incorporated polycaprolactone nanofibers (PD-PCL nanofibers)	Sustained release and osteogenic differentiation ↑; mesenchymal stem-cell viability maintained	[77]

Note: Route/regimen and comparator details are reported in Table 3, Table 4 and Table 5; study-level evidence appraisals are provided in Supplementary Table S4. Polydatin-specific attribution requires component-specific controls in multicomponent formulations. Upward (↑) and downward (↓) arrows indicate increases and decreases, respectively.

## Data Availability

No new data were created or analyzed in this study. Data sharing is not applicable to this article.

## Supplementary Materials

The following supporting information can be downloaded at: https://www.mdpi.com/article/10.3390/pharmaceutics18091134/s1, Table S1: PubMed search strategy and query-specific yields; Table S2: Screening decisions for records identified in the 26 August 2026 updates [62,90,91,126,127]; Table S3: Review-specific evidence-classification framework; Table S4: Author appraisal of the evidence level and targeting basis of included polydatin formulation and delivery studies [21,22,23,24,25,29,37,65,66,67,68,69,70,71,72,73,74,75,76,77,78,79,80,81,84,85,86,87,88,89,90,91,92,93,128,129,130,131]; Figure S1: Adapted PRISMA-style flow of literature identification and evidence-map screening. Seven complementary PubMed searches were conducted on 7 July 2026 and updated on 26 August 2026. The combined searches yielded 590 record occurrences. After removal of 156 duplicate occurrences by PMID, 434 unique records were screened by title and abstract; 277 were excluded using one mutually exclusive primary reason per record, and 157 were retained for synthesis. Retained evidence comprised 38 core delivery/formulation records, 31 supporting formulation/pharmacokinetic records, and 88 mechanistic/background records. The three evidence streams were synthesized separately and should not be interpreted as a homogeneous intervention dataset. PK, pharmacokinetics; QC, quality control; Table S5: Complete study-level evidence for the pharmacological mechanisms of polydatin [10,11,12,37,38,40,41,42,43,44,45,46,51,52,54,56,59,61,132,133,134].
