# Supplementary material for: Polydatin Delivery Systems and Nanomedicine: Pharmacology, Preclinical Evidence, and Translation
Source: Pharmaceutics. 2026 Sep 9;18(9):1134. doi: 10.3390/pharmaceutics18091134 (PMC13610665; doi:10.3390/pharmaceutics18091134)
Supplement: Supplementary file 1 [file pharmaceutics-18-01134-s001.zip › pharmaceutics-4524175-supplementary.pdf]

Supplementary Materials

Polydatin Delivery Systems and Nanomedicine: Pharmacology, Preclinical Evidence, and Translation

Supplementary Table S1. PubMed search strategy and query-specific yields

The initial searches were conducted on 7 July 2026. The identical seven queries were updated on 26 August 2026 using the PubMed Entrez Date (EDAT) window 8 July–26 August 2026. Counts are record occurrences before cross-query deduplication.

ID	Focus	Exact PubMed query	Initial yield	Update yield	Combined occurrences
**S1**	Broad polydatin/piceid delivery search	(<br>polydatin[tiab]<br>OR piceid[tiab]<br>OR "resveratrol glucoside"[tiab]<br>OR "resveratrol-3-O-glucoside"[tiab]<br>OR "3,4',5-trihydroxystilbene-3-beta-D-glucoside"[tiab]<br>)<br>AND<br>(<br>nanoparticle*[tiab]<br>OR nanocarrier*[tiab]<br>OR nanodelivery[tiab]<br>OR "nano-delivery"[tiab]<br>OR nanomedicine[tiab]<br>OR nanoformulation*[tiab]<br>OR "nano formulation"[tiab]<br>OR "drug delivery"[tiab]<br>OR "Drug Delivery Systems"[Mesh]<br>OR "Nanoparticles"[Mesh]<br>OR liposome*[tiab]<br>OR "Liposomes"[Mesh]<br>OR micelle*[tiab]<br>OR "Micelles"[Mesh]<br>OR polymeric nanoparticle*[tiab]<br>OR PLGA[tiab]<br>OR "poly(lactic-co-glycolic acid)"[tiab]<br>OR chitosan[tiab]<br>OR "Chitosan"[Mesh]<br>OR alginate[tiab]<br>OR exosome*[tiab]<br>OR "Exosomes"[Mesh]<br>OR biomimetic[tiab]<br>OR "cell membrane"[tiab]<br>OR "metal-organic framework*"[tiab]<br>OR MOF[tiab]<br>OR "solid lipid nanoparticle*"[tiab]<br>OR "nanostructured lipid carrier*"[tiab]<br>OR nanoemulsion*[tiab]<br>OR cyclodextrin*[tiab]<br>OR hydrogel*[tiab]<br>)	61	2	63
**S2**	High-specificity core formulation/delivery search	(<br>polydatin[tiab]<br>OR piceid[tiab]<br>OR "trans-piceid"[tiab]<br>OR "trans piceid"[tiab]<br>OR "resveratrol glucoside"[tiab]<br>OR "resveratrol-3-O-glucoside"[tiab]<br>OR "resveratrol 3-O-glucoside"[tiab]	33	2	35

ID	Focus	Exact PubMed query	Initial yield	Update yield	Combined occurrences
		)<br>AND<br>(<br>formulation*[tiab]<br>OR "drug delivery"[tiab]<br>OR "delivery system*"[tiab]<br>OR carrier*[tiab]<br>OR encapsulat*[tiab]<br>OR loaded[tiab]<br>OR loading[tiab]<br>OR incorporat*[tiab]<br>OR complex*[tiab]<br>OR "inclusion complex*"[tiab]<br>OR release[tiab]<br>OR "sustained release"[tiab]<br>OR "controlled release"[tiab]<br>OR bioavailability[tiab]<br>OR pharmacokinetic*[tiab]<br>OR solubility[tiab]<br>OR stability[tiab]<br>)<br>AND<br>(<br>nanoparticle*[tiab]<br>OR nanocarrier*[tiab]<br>OR nanomedicine[tiab]<br>OR nanoformulation*[tiab]<br>OR nanocapsule*[tiab]<br>OR liposome*[tiab]<br>OR micelle*[tiab]<br>OR PLGA[tiab]<br>OR "poly(lactic-co-glycolic acid)"[tiab]<br>OR chitosan[tiab]<br>OR cyclodextrin*[tiab]<br>OR hydrogel*[tiab]<br>OR nanofiber*[tiab]<br>OR scaffold*[tiab]<br>OR exosome*[tiab]<br>OR "extracellular vesicle*"[tiab]<br>OR "metal-organic framework*"[tiab]<br>OR MOF[tiab]<br>OR ZIF-8[tiab]<br>OR nanozyme*[tiab]<br>OR "gold nanoparticle*"[tiab]<br>OR "graphene oxide"[tiab]<br>)<br>NOT<br>(<br>review[Publication Type]<br>OR "Systematic Review"[Publication Type]<br>OR meta-analysis[Publication Type]<br>OR editorial[Publication Type]<br>OR letter[Publication Type]<br>OR comment[Publication Type]<br>OR "Retracted Publication"[Publication Type]<br>OR "Retraction of Publication"[Publication Type]<br>)<br>NOT<br>(<br>Vitis[tiab]<br>OR grapevine[tiab]<br>OR grape[tiab]<br>OR wine[tiab]			

ID	Focus	Exact PubMed query	Initial yield	Update yield	Combined occurrences
		OR "plant cell culture*"[tiab]<br>OR "suspension-cultured cell*"[tiab]<br>OR elicitor*[tiab]<br>OR phytoalexin*[tiab]<br>OR biosynthesis[tiab]<br>)			
**S3**	High-recall formulation/delivery gap search	(<br>polydatin[tiab]<br>OR piceid[tiab]<br>OR "trans-piceid"[tiab]<br>OR "trans piceid"[tiab]<br>OR "trans-polydatin"[tiab]<br>OR "trans polydatin"[tiab]<br>OR "resveratrol glucoside"[tiab]<br>OR "resveratrol-3-O-glucoside"[tiab]<br>OR "resveratrol 3-O-glucoside"[tiab]<br>)<br>AND<br>(<br>nanoparticle*[tiab]<br>OR nanocarrier*[tiab]<br>OR nanomedicine[tiab]<br>OR nanoformulation*[tiab]<br>OR nanocapsule*[tiab]<br>OR nanoliposome*[tiab]<br>OR liposome*[tiab]<br>OR micelle*[tiab]<br>OR PLGA[tiab]<br>OR "poly(lactic-co-glycolic acid)"[tiab]<br>OR PCL[tiab]<br>OR polycaprolactone[tiab]<br>OR chitosan[tiab]<br>OR cyclodextrin*[tiab]<br>OR "inclusion complex*"[tiab]<br>OR hydrogel*[tiab]<br>OR nanofiber*[tiab]<br>OR scaffold*[tiab]<br>OR dressing*[tiab]<br>OR exosome*[tiab]<br>OR "extracellular vesicle*"[tiab]<br>OR biomimetic[tiab]<br>OR "cell membrane"[tiab]<br>OR "metal-organic framework*"[tiab]<br>OR MOF[tiab]<br>OR ZIF-8[tiab]<br>OR nanozyme*[tiab]<br>OR "gold nanoparticle*"[tiab]<br>OR AuNP*[tiab]<br>OR "graphene oxide"[tiab]<br>OR formulation*[tiab]<br>OR "drug delivery"[tiab]<br>OR "delivery system*"[tiab]<br>OR carrier*[tiab]<br>OR encapsulat*[tiab]<br>OR loaded[tiab]<br>OR loading[tiab]<br>OR incorporat*[tiab]<br>OR release[tiab]<br>OR "sustained release"[tiab]<br>OR "controlled release"[tiab]<br>OR bioavailability[tiab]<br>OR pharmacokinetic*[tiab]<br>OR solubility[tiab]	180	3	183

ID	Focus	Exact PubMed query	Initial yield	Update yield	Combined occurrences
		OR stability[tiab]<br>)<br>NOT<br>(<br>review[Publication Type]<br>OR "Systematic Review"[Publication Type]<br>OR meta-analysis[Publication Type]<br>OR editorial[Publication Type]<br>OR letter[Publication Type]<br>OR comment[Publication Type]<br>OR "Retracted Publication"[Publication Type]<br>OR "Retraction of Publication"[Publication Type]<br>)<br>NOT<br>(<br>Vitis[tiab]<br>OR grapevine[tiab]<br>OR grape[tiab]<br>OR wine[tiab]<br>OR "plant cell culture*"[tiab]<br>OR "suspension-cultured cell*"[tiab]<br>OR elicitor*[tiab]<br>OR phytoalexin*[tiab]<br>OR biosynthesis[tiab]<br>)			
**S4**	Solid-state, film, and microneedle strategies	(<br>polydatin[tiab]<br>OR piceid[tiab]<br>OR "trans-piceid"[tiab]<br>OR "trans-polydatin"[tiab]<br>OR "resveratrol glucoside"[tiab]<br>OR "resveratrol-3-O-glucoside"[tiab]<br>)<br>AND<br>(<br>"hot melt extrusion"[tiab]<br>OR "hot-melt extrusion"[tiab]<br>OR HME[tiab]<br>OR "co-crystal*"[tiab]<br>OR cocrystal*[tiab]<br>OR "co crystal*"[tiab]<br>OR "solid dispersion*"[tiab]<br>OR "amorphous solid dispersion*"[tiab]<br>OR film*[tiab]<br>OR "oral film*"[tiab]<br>OR "buccal film*"[tiab]<br>OR "polymeric film*"[tiab]<br>OR "fast dissolving film*"[tiab]<br>OR "mucoadhesive film*"[tiab]<br>OR microneedle*[tiab]<br>OR "micro needle*"[tiab]<br>OR patch*[tiab]<br>)<br>NOT<br>(<br>review[Publication Type]<br>OR "Systematic Review"[Publication Type]<br>OR meta-analysis[Publication Type]<br>OR editorial[Publication Type]<br>OR letter[Publication Type]<br>OR comment[Publication Type]<br>OR "Retracted Publication"[Publication Type]<br>OR "Retraction of Publication"[Publication Type]	18	0	18

ID	Focus	Exact PubMed query	Initial yield	Update yield	Combined occurrences
		)			
**S5**	PK, bioavailability, biodistribution, and analytical methods	(<br>polydatin[tiab]<br>OR piceid[tiab]<br>OR "trans-piceid"[tiab]<br>OR "trans-polydatin"[tiab]<br>OR "resveratrol glucoside"[tiab]<br>OR "resveratrol-3-O-glucoside"[tiab]<br>)<br>AND<br>(<br>pharmacokinetic*[tiab]<br>OR bioavailability[tiab]<br>OR biodistribution[tiab]<br>OR "tissue distribution"[tiab]<br>OR absorption[tiab]<br>OR metabolism[tiab]<br>OR "plasma concentration"[tiab]<br>OR "plasma concentrations"[tiab]<br>OR Cmax[tiab]<br>OR Tmax[tiab]<br>OR AUC[tiab]<br>OR "half-life"[tiab]<br>OR "LC-MS"[tiab]<br>OR "LC-MS/MS"[tiab]<br>OR "UPLC-MS/MS"[tiab]<br>OR "UHPLC"[tiab]<br>OR "HPLC"[tiab]<br>)<br>NOT<br>(<br>review[Publication Type]<br>OR "Systematic Review"[Publication Type]<br>OR meta-analysis[Publication Type]<br>OR editorial[Publication Type]<br>OR letter[Publication Type]<br>OR comment[Publication Type]<br>OR "Retracted Publication"[Publication Type]<br>OR "Retraction of Publication"[Publication Type]<br>)	185	1	186
**S6**	Mechanistic gap search: inflammasome, STING, ferroptosis, pyroptosis	(<br>polydatin[tiab]<br>OR piceid[tiab]<br>OR "trans-piceid"[tiab]<br>OR "trans-polydatin"[tiab]<br>OR "resveratrol glucoside"[tiab]<br>)<br>AND<br>(<br>NLRP3[tiab]<br>OR inflammasome[tiab]<br>OR "cGAS-STING"[tiab]<br>OR cGAS[tiab]<br>OR STING[tiab]<br>OR ferroptosis[tiab]<br>OR ferroptotic[tiab]<br>OR pyroptosis[tiab]<br>OR pyroptotic[tiab]<br>OR "GSDMD"[tiab]<br>OR "gasdermin D"[tiab]<br>OR "caspase-1"[tiab]<br>OR "Caspase 1"[tiab]<br>OR "IL-1β"[tiab]	97	1	98

ID	Focus	Exact PubMed query	Initial yield	Update yield	Combined occurrences
		OR "IL-1beta"[tiab]<br>OR "IL-18"[tiab]<br>OR "ASC speck"[tiab]<br>)<br>NOT<br>(<br>review[Publication Type]<br>OR "Systematic Review"[Publication Type]<br>OR meta-analysis[Publication Type]<br>OR editorial[Publication Type]<br>OR letter[Publication Type]<br>OR comment[Publication Type]<br>OR "Retracted Publication"[Publication Type]<br>OR "Retraction of Publication"[Publication Type]<br>)			
**S7**	Nano-delivery plus mechanistic terms	(<br>polydatin[tiab]<br>OR piceid[tiab]<br>OR "resveratrol glucoside"[tiab]<br>)<br>AND<br>(<br>nanoparticle*[tiab]<br>OR nanocarrier*[tiab]<br>OR nanomedicine[tiab]<br>OR nanoformulation*[tiab]<br>OR liposome*[tiab]<br>OR micelle*[tiab]<br>OR chitosan[tiab]<br>OR PLGA[tiab]<br>OR hydrogel*[tiab]<br>OR exosome*[tiab]<br>OR nanozyme*[tiab]<br>OR formulation*[tiab]<br>OR delivery[tiab]<br>)<br>AND<br>(<br>NLRP3[tiab]<br>OR inflammasome[tiab]<br>OR "cGAS-STING"[tiab]<br>OR ferroptosis[tiab]<br>OR pyroptosis[tiab]<br>OR "GSDMD"[tiab]<br>OR "caspase-1"[tiab]<br>OR "IL-1β"[tiab]<br>OR "IL-18"[tiab]<br>)<br>NOT<br>(<br>review[Publication Type]<br>OR "Systematic Review"[Publication Type]<br>OR meta-analysis[Publication Type]<br>OR "Retracted Publication"[Publication Type]<br>OR "Retraction of Publication"[Publication Type]<br>)	7	0	7

Note: The combined searches produced 590 record occurrences. After removal of 156 duplicate occurrences, 434 unique records were screened. The update contributed five new unique records (three retained and two excluded).

Supplementary Table S2. Screening decisions for records identified in the 26 August 2026 updates [62,90,91,126,127]

PMID	Search set(s)	Record	Decision	Evidence stream/class	Formal reason or evidence boundary
**42583147** [85]	S1; S2; S3	Biocompatible chitosan-coated polydatin@ZIF-8 MOF nanostructures: synthesis, drug delivery and enhanced antibacterial properties. RSC advances; DOI: 10.1039/d6ra04037c	**Include**	Core delivery/formulation; F1+B1; T0	Polydatin was encapsulated in a chitosan-coated ZIF-8 nanocarrier; formulation characterization, drug loading, release, and antibacterial testing were reported. No in-vivo exposure, efficacy, or selective targeting evidence was reported.
**42487236** [88]	S1; S2; S3	A Binary Natural Herbal Co-Assembled Hydrogel for Expediting MRSA-Infected Diabetic Wound Healing via Robust Sterilization, Anti-Inflammation and Tissue Repair. Advanced healthcare materials; DOI: 10.1002/adhm.71468	**Include**	Core delivery/formulation; F1+B1+E1; T0	Polydatin was a defined component of a mangiferin-polydatin co-assembled hydrogel with release, antibacterial/biofilm, and diabetic-rat wound outcomes. Effects are attributable to the complete binary formulation because component-specific treatment controls were not reported in the abstract.
**42468884** [62]	S6	Polydatin improves mitochondrial dynamics dysrhythmia by regulating BMAL1 to prevent and treat metabolic dysfunction-associated fatty liver disease. Journal of ethnopharmacology; DOI: 10.1016/j.jep.2026.122195	**Include**	Mechanistic/background; Direct mechanism/background	Free-polydatin experiments used BMAL1 knockdown in hepatic cells and a high-fat-diet rat model to test mitochondrial-dynamics and mitophagy-related mechanism; retained only as delivery-rationale background.
**42595105** [126]	S3	Polydatin in combination with hawthorn flavonoids alleviates atherosclerosis by inhibiting mitochondrial division through the YTHDF2-DRP1 axis. Journal of advanced research; DOI: 10.1016/j.jare.2026.08.037	**Exclude**	Multicomponent extract/formula/combination	The intervention combined polydatin with hawthorn flavonoids, was not a defined delivery system, and did not permit attribution of the reported mechanism or efficacy to polydatin delivery.
**42413793** [127]	S5	Integrated prioritization of candidate quality markers for Smilacis Chinae Rhizoma under the five-principle framework through chemical profiling, effectiveness evaluation, and bionic sensing. Journal of ethnopharmacology; DOI: 10.1016/j.jep.2026.122170	**Exclude**	Botanical/food/extraction/production/QC	The study prioritized quality markers for Smilacis Chinae Rhizoma; polydatin was a candidate marker rather than a defined pharmaceutical payload or delivery intervention.

Supplementary Table S3. Review-specific evidence-classification framework

These codes and grades are operational categories developed by the authors for consistent appraisal in this review; they are not a previously validated external scale. The framework was informed by minimum reporting recommendations for bio-nano studies and by experimental evidence that fluorescent-label distribution may not represent intact-carrier or payload distribution (PMID: 30190620; PMID: 34452106). Po, polydatin.

Code	Definition	What it permits	What it does not permit
F0	Formulation/physicochemical characterization only	Size, morphology, loading or structural statements actually measured	Bioavailability, efficacy, or targeting
F1	In-vitro release, stability, dissolution, or permeation	Pharmaceutical-performance claim in the tested model	Improved in-vivo bioavailability without exposure data
B1	In-vitro or ex-vivo biological activity	Cell/tissue-model efficacy	In-vivo therapeutic superiority
E1	In-vivo efficacy/tolerability without quantified exposure	Model-specific efficacy with comparator stated	PK, tissue exposure, or targeting
PK1	Direct within-study plasma PK: same route and nominal dose, free vs formulated Po	Formulation-associated exposure difference	Cross-study generalization or organ targeting
D1	Carrier/surrogate distribution (dye, labeled siRNA, peptide/phage)	Carrier localization only	Distribution of polydatin itself
D2	Direct quantitative polydatin concentration in tissue	Drug distribution/enrichment at measured times	Active targeting without matched non-targeted control/mechanism
M1	Mechanism-supported carrier uptake/targeting with blocking, competition, or appropriate negative controls	Mechanistic carrier-level targeting	Polydatin targeting unless drug itself is quantified
T0	No targeting evidence	No targeting wording	Any targeted-delivery claim
T1	Localization/distribution only	‘localized’, ‘distributed’, or ‘enriched’ with analyte stated	‘actively targeted’ or ‘selective’
T2	Mechanism-supported carrier targeting	Carrier/siRNA/dye targeting with model and controls stated	Polydatin targeting if Po was not quantified
T3	Quantitative tissue Po plus matched non-targeted comparator and mechanism	Drug-level active-targeting claim	None of the audited current studies reached T3

Supplementary Table S4. Author appraisal of the evidence level and targeting basis of included polydatin formulation and delivery studies [21–25,29,37,65–81,84–93,128–131]

The table reports the maximum conclusion supported by each study rather than an assessment of general study quality. T0 indicates no targeting evidence; T1, localization/distribution only; T2, mechanism-supported carrier targeting; and T3, quantitative tissue polydatin plus a matched non-targeted comparator and mechanistic evidence. No study reached T3.

PMID	Year	Platform/formulation	Evidence code	Target grade	Verification status	Maximum supported conclusion	Key limitation
37466428 [22]	2023	Exosome-coated/biomimetic nanoparticle	F0+B1+E1+D1	T1	Full text verified	In-vivo efficacy plus exosome-carrier localization	No PK/direct tissue Po; exosome independently active
40521186 [80]	2025	Targeted LNP / co-delivery	F1+B1+E1+D1+M1	T2	Full text verified	AEC-directed carrier/siRNA delivery in VILI	No Po PK or tissue Po; intratracheal route
39392049 [81]	2024	PLGA/polymeric nanoparticle	F1+B1+D1+M1	T2	Full text verified	CD44-dependent carrier uptake in A549 cells	In-vitro RhB surrogate only
41189670 [76]	2025	Fucoidan/polysaccharide targeting system	F1+B1+E1+PK1+D1+D2+M1	T2	Full text verified	Improved oral exposure; carrier-level dual-targeting mechanism	n=3; species/time discordance; active 4-PBA
39657878 [75]	2025	Chitosan-based nanocarrier	F1+B1+E1+PK1+D1+D2	T1	Full text verified	Improved oral exposure and multi-organ Po enrichment	n=3; one tissue time; no active-target proof
35745646 [86]]	2022	PCL/polymeric platform	F1+B1	T0	Title/abstract-level classification; not used for detailed quantitative claims	Nanofiber characterization and osteogenic cell response	No in-vivo exposure or targeting

PMID	Year	Platform/formulation	Evidence code	Target grade	Verification status	Maximum supported conclusion	Key limitation
41796779 [77]	2026	Hydrogel/local sustained-release system	F1+B1+E1	T0	Title/abstract-level classification; not used for detailed quantitative claims	Local hydrogel delivery and model-specific efficacy	Evidence-map level; verify detailed comparators
35964388 [25]	2022	Scaffold/dressing/local delivery	F1+B1+E1	T0	Title/abstract-level classification; not used for detailed quantitative claims	Scaffold performance and wound-model efficacy	Local multicomponent system; no PK
41302457 [83]	2025	Chitosan-based nanocarrier	F1+B1	T0	Title/abstract-level classification; not used for detailed quantitative claims	Nanocapsule formulation/release and in-vitro performance	No verified in-vivo exposure
25848217 [67]	2015	Liposome/lipid system	F1+PK1	T0	Full text verified	Direct evidence of increased oral systemic exposure	No tissue targeting; internal route-caption error
25916244 [65]	2015	Cyclodextrin/supramolecular complex	F0	T0	Title/abstract-level classification; not used for detailed quantitative claims	Cyclodextrin inclusion-complex structure	No in-vivo bioavailability
35541971 [29]	2018	Cyclodextrin/supramolecular complex	F0+B1	T0	Title/abstract-level classification; not used for detailed quantitative claims	Photostability and in-vitro antioxidant performance	No in-vivo bioavailability
41218521 [84]	2026	PCL/polymeric platform	F1+B1	T0	Title/abstract-level classification; not used for detailed quantitative claims	Polymeric formulation and radioresistant-cell response	Targeting in title is not organ targeting
38160781 [74]	2024	Mechanistic support	F1+B1+E1	T0	Title/abstract-level classification; not used for detailed quantitative claims	Formulation plus intestinal-toxicity efficacy	No verified exposure/targeting
33520856 [72]	2020	Chitosan-based nanocarrier	F1+E1	T0	Title/abstract-level classification; not used for detailed quantitative claims	Chitosan-NP characterization and diabetic-nephropathy efficacy	No quantified PK or renal Po
31176102 [82]	2019	PK/metabolism/analytical support	F0+B1	T0	Title/abstract-level classification; not used for detailed quantitative claims	Free versus nanocapsulated Po in organotypic culture	Ex-vivo only
31758992 [21]	2020	Chitosan-based nanocarrier	F1+B1+E1	T0	Title/abstract-level classification; not used for detailed quantitative claims	Formulation plus diabetes efficacy	No verified PK or tissue drug
33832416 [71]	2021	Chitosan-based nanocarrier	F1+E1	T0	Title/abstract-level classification; not used for detailed quantitative claims	Formulated Po in diabetic-rat liver outcomes	No verified PK or liver Po
42235587 [87]	2026	Hydrogel/local sustained-release system	F1+B1+E1	T0	Title/abstract-level classification; not used for detailed quantitative claims	Local sustained release and wound healing	Multicomponent local hydrogel; no systemic PK
32274310 [23]	2020	Background/supporting evidence	F1+B1+E1+D1+D2	T1	Full text verified	Hepatic delivery and stimulus-responsive release	No disease-selective Po accumulation; route-confounded efficacy
42067091 [95]	2026	Hydrogel/local sustained-release system	F1+B1+E1	T0	Title/abstract-level classification; not used for detailed quantitative claims	Infection-responsive local hydrogel efficacy	Multicomponent nanozyme system; Po-specific attribution limited
31491872 [24]	2019	PLGA/polymeric nanoparticle	F1+B1+E1	T0	Title/abstract-level classification; not used for detailed quantitative claims	PLGA formulation and carcinogenesis-model efficacy	No verified PK/targeting
39786589 [79]	2025	Chitosan-based nanocarrier	F1+B1+E1	T0	Full text verified	In-vivo efficacy versus disease/blank-carrier controls	No free-Po comparator or exposure data
39820828 [73]	2025	Chitosan-based nanocarrier	F1+B1+E1	T0	Full text verified	Route/dose-matched efficacy over free Po	No PK/tissue Po; lung accumulation unverified
34339076 [78]	2021	Inorganic/metal/nanozyme-integrated system	F0+B1+E1	T0	Full text verified	Route-matched antitumor efficacy	No loading/release/distribution/targeting data

PMID	Year	Platform/formulation	Evidence code	Target grade	Verification status	Maximum supported conclusion	Key limitation
28069190 [68]	2017	Liposome/lipid system	F0+F1	T0	Title/abstract-level classification; not used for detailed quantitative claims	Liposome process optimization and encapsulation	No in-vivo exposure
36235550 [89]	2022	Cyclodextrin/supramolecular complex	F1+B1	T0	Title/abstract-level classification; not used for detailed quantitative claims	Dissolution/in-vitro permeability performance	Title question does not establish in-vivo bioavailability
37111336 [90]	2023	Electrospun nanofiber/local delivery	F1+B1	T0	Title/abstract-level classification; not used for detailed quantitative claims	Local tablet/nanofiber performance	Extract-based, no systemic PK
37760078 [128]	2023	Cyclodextrin/supramolecular complex	F1+B1	T0	Title/abstract-level classification; not used for detailed quantitative claims	Electrospun-system antioxidant performance	Resveratrol/extract context; no Po PK
39467101 [129]	2025	Background/supporting evidence	F0+B1	T0	Title/abstract-level classification; not used for detailed quantitative claims	Water-soluble conjugate and antibacterial/oxidation assays	Conjugate, not a validated drug-delivery PK system
34577193 [66]	2021	Co-crystal/solid-state strategy	F0+F1+B1	T0	Title/abstract-level classification; not used for detailed quantitative claims	Cocrystal structure, dissolution, and in-vitro cytotoxicity	Non-nano; no in-vivo bioavailability
37765035 [92]	2023	Hot-melt extrusion/solid dispersion	F1+B1	T0	Title/abstract-level classification; not used for detailed quantitative claims	Hot-melt-extrusion physicochemical/buccal performance	Non-nano; no in-vivo bioavailability
39769807 [91]	2024	Film/tablet/mucoadhesive formulation	F1+B1	T0	Title/abstract-level classification; not used for detailed quantitative claims	Film optimization and local-performance assays	Extract-based local system; no systemic exposure
32764930 [130]	2020	Inorganic/metal/nanozyme-integrated system	F0+B1+E1	T0	Title/abstract-level classification; not used for detailed quantitative claims	Multicomponent Au@AgNP wound-healing efficacy	Po-specific loading/release and attribution unclear
18024274 [131]	2007	Scaffold/dressing/local delivery	F0+E1	T0	Title/abstract-level classification; not used for detailed quantitative claims	Non-nano composite dressing efficacy	Boundary item; not a Po nanocarrier PK study
18420333 [37]	2008	Liposome/lipid system	B1	T0	Title/abstract-level classification; not used for detailed quantitative claims	Piceid antioxidant behavior in model liposomes	Liposomes are experimental membranes, not a drug formulation
42583147 [85]	2026	Chitosan-coated polydatin@ZIF-8 MOF nanostructure	F1+B1	T0	Title/abstract-level classification; not used for detailed quantitative claims	Formulation characterization, loading, release, and antibacterial activity	No in-vivo exposure, efficacy, or selective-targeting evidence
42487236 [88]	2026	Mangiferin-polydatin co-assembled hydrogel	F1+B1+E1	T0	Title/abstract-level classification; not used for detailed quantitative claims	Local release, antibacterial/biofilm activity, and diabetic-wound efficacy	Multicomponent system; component-specific attribution and exposure were not established

Figure S1

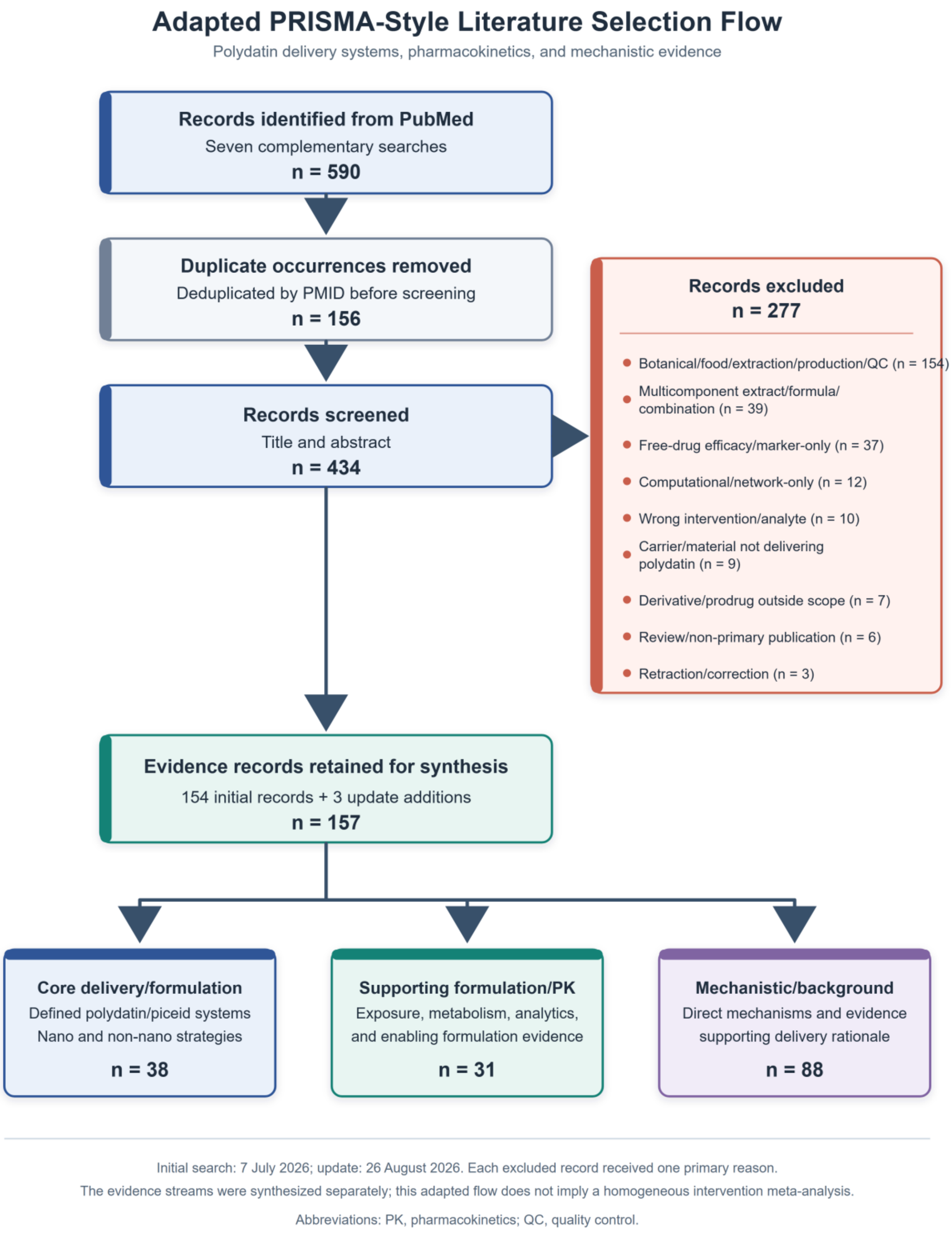

Figure S1. Adapted PRISMA-style flow of literature identification and evidence-map screening. Seven complementary PubMed searches were conducted on 7 July 2026 and updated on 26 August 2026. The combined searches yielded 590 record occurrences. After removal of 156 duplicate occurrences by PMID, 434 unique records were screened by title and abstract; 277 were excluded using one mutually exclusive primary reason per record, and 157 were retained for synthesis. Retained evidence comprised 38 core delivery/formulation records, 31 supporting formulation/pharmacokinetic records, and 88 mechanistic/background records. The three evidence streams were synthesized separately and should not be interpreted as a homogeneous intervention dataset. PK, pharmacokinetics; QC, quality control.

Supplementary Table S5. Complete study-level evidence for the pharmacological mechanisms of polydatin [10–12,37,38,40–46,51,52,54,56,59,61,132–134]

Model	Polydatin exposure	Comparator/mechanistic control	Evidence basis	Principal molecular change	Principal functional outcome	Study identifier
Cell-free: ABIP-induced linoleic-acid peroxidation in SDS micelles and DPPC liposomes	2 µM (peroxidation); 12.5-50 µM (DPPH)	Piceid; resveratrol; BHT; α-tocopherol	Chemical and membrane-biophysical assays	O2 consumption and DPPH radicals decreased; membrane partitioning was demonstrated	Membrane lipid peroxidation decreased	Fabris et al., 2008; PMID: 18420333 [37]
In vitro: primary rat cardiomyocytes; Ang II (100 nM, 24 h)	5-50 µM; 1-h pretreatment	Control; Ang II; polydatin; Ang II + polydatin	Cellular dose-response; pathway association	NOX2/NOX4 and hypertrophy-associated ANF/beta-MHC decreased	ROS, cell size, and protein synthesis decreased	Zhang et al., 2015; PMID: 25488910 [38]
In vivo: rats; Ang II infusion (300 ng/kg/min, 28 d)	50 mg/kg/day, gavage; from 7 d before Ang II and throughout infusion	Sham; Ang II; polydatin; Ang II + polydatin	In-vivo dose-response; pathway association	NOX2/NOX4 and hypertrophy-associated ANF/beta-MHC decreased	Myocardial superoxide, hypertrophy, and fibrosis decreased	Zhang et al., 2015; PMID: 25488910 [38]
In vivo/ex vivo: rat hemorrhagic shock; isolated arterial smooth-muscle cells	30 mg/kg i.v.; after shock and before blood reinfusion; 120 min	Sham; shock; cyclosporin A; resveratrol; polydatin	Mitochondrial assays with cyclosporin A and resveratrol comparators	mPTP opening and lipid hydroperoxides decreased; ATP increased	Mitochondrial potential and ATP increased; mitochondrial swelling and lysosomal instability decreased	Wang et al., 2012; PMID: 22277937 [40]
In vitro: primary mouse chondrocytes; IL-1β stimulation	25 or 50 µM; approximately 24 h	Control; IL-1β; dexamethasone; graded polydatin	Cellular dose-response; pathway association	SIRT3/SOD2 and COL2 increased; beta-catenin and MMP-13 decreased	Mitochondrial ROS and matrix catabolism decreased	Mao et al., 2025; PMID: 40505483 [41]
In vivo: MIA- or DMM-induced osteoarthritis in mice	25 or 50 mg/kg/day i.p.; from day 3; 4 or 12 wk	Sham; model; low-dose polydatin; high-dose polydatin	Two in-vivo osteoarthritis models	SIRT3/SOD2 and COL2 increased; MMP-13 decreased	Cartilage degeneration and osteophyte formation decreased	Mao et al., 2025; PMID: 40505483 [41]
In vitro: fructose-exposed BRL-3A and HepG2 cells	10-40 µM; 24-48 h	Pioglitazone; miR-200a mimic; Keap1 siRNA; TXNIP siRNA; tBHQ; NAC	miR-200a mimic and Keap1/TXNIP knockdown	miR-200a/Nrf2 signaling increased; TXNIP/NLRP3 and lipogenic signaling decreased	ROS and intracellular lipid accumulation decreased	Zhao et al., 2018; PMID: 30014902 [42]
In vivo: rats; 10% fructose feeding	7.5-30 mg/kg/day, gavage for 7 wk after 6-wk fructose feeding	Control; fructose; graded polydatin; pioglitazone	In-vivo dose-response with pioglitazone comparator	miR-200a/Nrf2 signaling increased; TXNIP/NLRP3, TNF-alpha, and lipogenic signaling decreased	Steatosis and hepatic lipid deposition decreased	Zhao et al., 2018; PMID: 30014902 [42]
In vitro/cell-free: IgE/antigen-stimulated RBL-2H3 cells and rat mast cells; Lyn/Syk kinase assays	10-40 µM; pretreatment before antigen	Control; IgE/antigen; graded polydatin; Lyn/Syk kinase assays	Cell-free Lyn/Syk kinase assays plus cellular signaling	Lyn/Syk and downstream PI3K/Akt, MAPK, and NF-κB signaling decreased; Nrf2/HO-1 increased	Mast-cell degranulation, histamine release, and Ca2+ mobilization decreased	Ye et al., 2017; PMID: 28928455 [43]
In vivo: passive cutaneous and systemic anaphylaxis in mice	15, 30 or 40 mg/kg i.p.; 1 h before antigen	Normal; polydatin; IgE/antigen; graded polydatin	In-vivo dose-response	Histamine decreased	Vascular leakage and ear swelling decreased	Ye et al., 2017; PMID: 28928455 [43]
In vitro: LPS-stimulated BV2 microglia (0.5 µg/mL, 24 h)	2-8 µM; 1-h pretreatment	Control; LPS; graded polydatin	Lipid-raft fractionation and signaling association	Lipid-raft cholesterol and PI3K/Akt/NF-κB signaling decreased; ABCA1/ABCG1 increased	Lipid-raft formation and microglial activation decreased	Sun et al., 2021; PMID: 33509007 [44]
In vivo/ex vivo: CLP-induced sepsis-associated encephalopathy; isolated cortical neurons	45 mg/kg, 30 min before CLP; route NR; assessed to 48 h	Sham; CLP; polydatin; Ex527; SB203580; Ex527 + polydatin	SIRT1 and p38 inhibitor controls	SIRT1 increased; p38 activation and inflammatory cytokines decreased	Mitochondrial function improved and neuronal injury decreased	Huang et al., 2022; PMID: 35447391 [45]
Ex vivo: neutrophils from healthy donors and patients with SLE; spontaneous or PMA-induced NETs	100 µg/mL; 1-h pretreatment	Healthy and SLE neutrophils; vehicle; PMA; PMA + polydatin	Primary human neutrophils; ex-vivo association	ROS and extracellular DNA decreased	NET formation decreased	Liao et al., 2018; PMID: 30419963 [46]
In vivo: pristane-induced and MRL/lpr lupus models	45 mg/kg/day i.p.; 16 or 8 wk	Normal; model; polydatin; cyclophosphamide; mycophenolate mofetil	Two lupus models with active comparators	Autoantibodies and renal IgG deposition decreased	Renal NET deposition, proteinuria, and injury decreased	Liao et al., 2018; PMID: 30419963 [46]
In vitro: high-glucose rat glomerular mesangial cells (30 mM, 24 h)	40 µM; 24 h; 2 h for CETSA	C-176; STING siRNA; STING overexpression; proteasome inhibition	SPR/CETSA binding plus STING knockdown and overexpression	Polydatin-STING binding and STING ubiquitination were detected; TBK1/NF-κB and matrix markers decreased	Extracellular-matrix accumulation decreased	Liang et al., 2024; PMID: 38885772 [132]

Model	Polydatin exposure	Comparator/mechanistic control	Evidence basis	Principal molecular change	Principal functional outcome	Study identifier
In vivo: STZ-HFD diabetic mice with renal fibrosis	100 or 200 mg/kg/day, gavage for 10 wk	Control; diabetes; graded polydatin; C-176	In-vivo comparison with the STING inhibitor C-176	STING/TBK1/NF-κB signaling and matrix markers decreased	Glomerular injury and matrix deposition decreased	Liang et al., 2024; PMID: 38885772 [132]
In vivo: potassium-oxonate hyperuricemia and renal inflammation in rats	25 or 50 mg/kg/day, gavage for 7 d	Control; model; allopurinol; graded polydatin	Dose-response with allopurinol comparator	AMPK/SIRT1 increased; NF-κB/NLRP3/caspase-1 signaling decreased	Hyperuricemia and renal injury decreased	Chen et al., 2017; PMID: 28428988 [51]
In vitro: hyperosmolar-stressed human conjunctival epithelial cells (528 mOsm)	0.1-10 μM; 1-24 h	Control; hyperosmotic medium; graded polydatin; NAC	Cellular dose-response with NAC comparator	NF-κB/NLRP3/caspase-1 signaling decreased	ROS, cytotoxicity, and inflammasome activation decreased	Park et al., 2019; PMID: 31731792 [52]
In vivo/local: lacrimal-gland-excision dry-eye model in rats	0.05% or 0.5% eye drops; three times/day, postoperative days 3-7	Normal; vehicle; two polydatin concentrations	Topical dose-response in vivo	NLRP3 and inflammatory cytokines decreased; MUC5AC increased	Tear-film function and goblet-cell preservation increased	Park et al., 2019; PMID: 31731792 [52]
In vitro/biophysical: BMDMs, THP-1 and HEK293T cells; purified HSP90α; LPS plus ATP, MSU or nigericin	Usually 1-10 μM; 1-h pretreatment	Geldanamycin; MCC950; HSP90α knockout; HSP90β and AIM2 controls	Biophysical binding, HSP90α knockout, and inflammasome controls	HSP90α binding was detected; HSP90α ATPase activity and NLRP3-complex stability decreased	NLRP3-complex stability and pyroptotic signaling decreased	Yang et al., 2024; PMID: 39691768 [11]
In vivo: cerulein-induced acute pancreatitis in mice	5-20 mg/kg, gavage; 1 h before cerulein	Saline; model; graded polydatin; geldanamycin	In-vivo comparison with an HSP90 inhibitor	NLRP3/caspase-1/IL-1beta signaling decreased	Pancreatic and pulmonary injury decreased	Yang et al., 2024; PMID: 39691768 [11]
In vitro: uric-acid-stimulated human renal tubular epithelial cells with or without LPS	75 μg/mL; 24 h	Control; uric acid; polydatin; LPS + uric acid; LPS + uric acid + polydatin	Cellular marker association	NLRP3/caspase-1/GSDMD signaling decreased	Membrane permeabilization and cell death decreased	Shi et al., 2023; PMID: 36328529 [54]
In vivo: hypoxanthine/potassium-oxonate gouty nephropathy in mice	50 mg/kg/day for 14 d; route NR	Control; model; polydatin	In-vivo marker association	NLRP3/caspase-1/GSDMD signaling decreased	Renal inflammation, fibrosis, and injury decreased	Shi et al., 2023; PMID: 36328529 [54]
In vitro: hemin-treated Neuro2A cells (10 μM, 12 h)	60 μM during hemin exposure	Control; hemin; polydatin; ferrostatin-1	Ferrostatin-1 comparator	Iron accumulation and lipid peroxidation decreased; selenium-dependent GPx activity increased	Iron accumulation, lipid peroxidation, and cell injury decreased	Huang et al., 2021; PMID: 33839410 [56]
In vivo: controlled-cortical-impact traumatic brain injury in mice	50 mg/kg i.p.; once, 30 min after injury	Sham; model; polydatin	Post-injury in-vivo association	Iron deposition and lipid peroxidation decreased; selenium-dependent GPx activity increased	Lesion volume and neurological deficits decreased	Huang et al., 2021; PMID: 33839410 [56]
In vitro: HEK293T GPX4 reporter cells and MIN6 β-cells; palmitate under high glucose	Reporter: 0-20 μM for 24 h; MIN6: 5 or 10 μM, 8-h pretreatment plus 24-h insult	Reporter controls; palmitate/high glucose; erastin; graded polydatin	Promoter reporter and expression analysis	Nrf2/SLC7A11/GPX4 increased; ACSL4 and iron-uptake markers decreased	Beta-cell viability increased	Prasad et al., 2026; PMID: 41461268 [12]
In vitro: ox-LDL-stimulated human aortic endothelial cells (100 μg/mL, 24 h)	40 μM; 24 h	Ferrostatin-1; erastin; control siRNA; Nrf2 siRNA	Nrf2 knockdown plus ferroptosis controls	Nrf2/GPX4/SLC7A11 increased; inflammatory adhesion markers decreased	Lipid ROS and endothelial dysfunction decreased	Wu et al., 2025; PMID: 40886948 [133]
In vivo: HFD-fed ApoE-knockout mice with atherosclerosis	50 or 100 mg/kg/day, gavage for 8 wk	Wild type; model; graded polydatin	In-vivo dose-response	Nrf2/GPX4/SLC7A11 increased; TNF-alpha and VCAM-1 decreased	Plaque burden, inflammation, and ferroptosis-associated injury decreased	Wu et al., 2025; PMID: 40886948 [133]
In vitro: LPS-stimulated H9c2 cells (1 μg/mL, 24 h)	100 μM; 30-min pretreatment	Control; LPS; 3-MA; polydatin + 3-MA; SIRT6 siRNA	SIRT6 knockdown and autophagy inhibition	SIRT6 and autophagy markers increased; apoptotic and inflammatory signaling decreased	Autophagy increased; apoptosis and inflammation decreased	Yuan et al., 2020; PMID: 32394287 [59]
In vivo: CLP-induced septic cardiomyopathy in rats	30 mg/kg i.p.; 6 h before CLP; assessed at 48 h	Sham; CLP; 3-MA; polydatin + 3-MA	Autophagy inhibition in vivo	SIRT6 and autophagy markers increased; apoptotic and inflammatory signaling decreased	Cardiac dysfunction decreased	Yuan et al., 2020; PMID: 32394287 [59]
In vivo: subarachnoid-hemorrhage early brain injury in mice	10-30 mg/kg i.p.; 1 h after hemorrhage	Vehicle; graded polydatin; thapsigargin; sh-SIRT1	SIRT1 knockdown and ER-stress induction	SIRT1 increased; PERK/eIF2alpha/ATF4/CHOP and apoptotic signaling decreased	Brain edema, BBB disruption, and neuronal injury decreased	Han et al., 2024; PMID: 39295935 [10]

**Model**	**Polydatin exposure**	**Comparator/mechanistic control**	**Evidence basis**	**Principal molecular change**	**Principal functional outcome**	**Study identifier**
**In vitro: euploid and trisomy-21 human skin fibroblasts**	**20 µM; 24 h**	**Euploid ± polydatin; trisomy 21 ± polydatin**	**Euploid/trisomy-21 comparison; pathway association**	**miR-155 decreased; TFAM/CBL/BAG5/PINK1 increased**	**Bioenergetics and mitophagy-related responses increased; ROS, DNA damage, and senescence decreased**	**Valenti et al., 2025; PMID: 40280316 [61]**
**In vivo: wild-type and Parkin-knockout mice; CLP-induced sepsis-associated AKI**	**30 mg/kg, tail vein; immediately after CLP; assessed at 12 h**	**Parkin knockout; mdivi-1; Ex527; vehicle controls**	**Parkin knockout plus SIRT1 and fission inhibitors**	**Mitochondrial Parkin redistribution increased; apoptotic and NLRP3 signaling decreased**	**Mitophagy-related function and ATP increased; apoptosis and renal injury decreased**	**Gao et al., 2020; PMID: 32131850 [134]**

Note: This table preserves the complete study-level experimental matrix formerly presented in main-text Table 1. Author/year/PMID identifiers are used so that final EndNote linking does not depend on provisional reference numbering. Abbreviations: ABIP, 2,2'-azobis[2-(2-imidazolin-2-yl)propane]; Ang II, angiotensin II; BBB, blood-brain barrier; CLP, cecal ligation and puncture; DMM, destabilization of the medial meniscus; DPPC, dipalmitoylphosphatidylcholine; DPPH, 2,2-diphenyl-1-picrylhydrazyl; HFD, high-fat diet; i.p., intraperitoneal; i.v., intravenous; MIA, monosodium iodoacetate; mPTP, mitochondrial permeability transition pore; NET, neutrophil extracellular trap; NR, not reported; SLE, systemic lupus erythematosus; STZ, streptozotocin; DeltaPsi_m, mitochondrial membrane potential.
